# Reinvestigating the Coughing Rat Model of Pertussis To Understand *Bordetella pertussis* Pathogenesis

**DOI:** 10.1128/IAI.00304-21

**Published:** 2021-11-16

**Authors:** Jesse M. Hall, Jason Kang, Sophia M. Kenney, Ting Y. Wong, Graham J. Bitzer, Claire O. Kelly, Caleb A. Kisamore, Dylan T. Boehm, Megan A. DeJong, M. Allison Wolf, Emel Sen-Kilic, Alexander M. Horspool, Justin R. Bevere, Mariette Barbier, F. Heath Damron

**Affiliations:** a Department of Microbiology, Immunology and Cell Biology, School of Medicine, West Virginia University, Morgantown, West Virginia, USA; b Vaccine Development Center, WVU Health Sciences Center, Morgantown, West Virginia, USA; Washington State University

**Keywords:** *Bordetella pertussis*, cough, DTaP, plethysmography, rat

## Abstract

Bordetella pertussis is a highly contagious bacterium that is the causative agent of whooping cough (pertussis). Currently, acellular pertussis vaccines (aP, DTaP, and Tdap) are used to prevent pertussis disease. However, it is clear that the aP vaccine efficacy quickly wanes, resulting in the reemergence of pertussis. Furthermore, recent work performed by the CDC suggest that current circulating strains are genetically distinct from strains of the past. The emergence of genetically diverging strains, combined with waning aP vaccine efficacy, calls for reevaluation of current animal models of pertussis. In this study, we used the rat model of pertussis to compare two genetically divergent strains Tohama 1 and D420. We intranasally challenged 7-week-old Sprague-Dawley rats with 10^8^ viable Tohama 1 and D420 and measured the hallmark signs/symptoms of *B. pertussis* infection such as neutrophilia, pulmonary inflammation, and paroxysmal cough using whole-body plethysmography. Onset of cough occurred between 2 and 4 days after *B. pertussis* challenge, averaging five coughs per 15 min, with peak coughing occurring at day 8 postinfection, averaging upward of 13 coughs per 15 min. However, we observed an increase of coughs in rats infected with clinical isolate D420 through 12 days postchallenge. The rats exhibited increased bronchial restriction following *B. pertussis* infection. Histology of the lung and flow cytometry confirm both cellular infiltration and pulmonary inflammation. D420 infection induced higher production of anti-*B. pertussis* IgM antibodies compared to Tohama 1 infection. The coughing rat model provides a way of characterizing disease manifestation differences between *B. pertussis* strains.

## INTRODUCTION

Whooping cough (pertussis) is a respiratory disease that is caused by the bacterium Bordetella pertussis. Pertussis is characterized by severe leukocytosis, bronchopneumonia, hypoglycemia, and paroxysmal cough (1, 2). During the catarrhal stage, *B. pertussis* colonizes the upper respiratory epithelium. Once colonization occurs, *B. pertussis* releases toxins such as pertussis toxin (PT) and adenylate cyclase toxin (ACT), as well as others. The catarrhal stage is followed by the paroxysmal stage, which is characterized by severe coughing episodes. Innate and adaptive immune responses result in clearance of the bacterium, allowing for the patient or host to convalesce. Despite extensive research, a full understanding of *B. pertussis* pathogenesis remains elusive for reasons, such as vaccine pressure, a lack of predictive models, and strain evolution (3). One of the major reasons for this gap in knowledge is the lack of a suitable animal model that shares common disease symptoms as seen in humans. Besides nonhuman primates, rats are the only other known model of pertussis that share similar coughing manifestation as seen in humans (4). As of now, the origin or the trigger of the cough has yet to be determined (5). The rat model of pertussis offers a potential animal model that can be used to evaluate *B. pertussis* pathogenesis.

In 1938, Hornibrook and Ashburn were the first to report that rats infected with *B. pertussis* induced cough-like paroxysms and bronchopneumonia, as seen in humans (6). Hornibrook and Ashburn found that young rats could be infected and the bacterium could be cultured from the lungs (6). Infected rats produced coughs that could be heard from a distance of 20 feet (6). Of the 31 infected rats, 26 that did not succumb to the infection had pathology indicative of inflammation in the lungs, most notably early neutrophil infiltration, followed by recruitment of mononuclear cells (6). The subsequent studies utilized intrabronchial inoculation of *B. pertussis* encased in agar beads and confirmed leukocytosis and paroxysmal cough by sound activated tape recorders from 5 to 21 days postchallenge (7, 8). Further development of the rat model led to the evaluation of PT-negative strains and the evaluation of coughs (9). Strain BP357, which is deficient in PT resulted in low cough induction (9). Further evaluation of the rat model demonstrated leukocytosis, weight loss, and paroxysmal cough in Sprague-Dawley rats during the course of *B. pertussis* infection (10). During the development phase of the acellular pertussis vaccine (aP and DTaP), the rat model was used to test vaccine efficacy of various acellular pertussis formulations and was used to validate protection against the onset of leukocytosis and cough (11). Recently, an intranasal rat model has been used to evaluate bacterial factors responsible for cough in Bordetella bronchiseptica (12). BspR, which is an anti-sigma factor of *B. bronchiseptica*, plays some role in cough induction upon infection (12). Collectively, these studies show that the pertussis rat model can be used to critically evaluate *B. pertussis* pathogenesis and disease progression.

In the 1940s, whole-cell pertussis (wP and DTP) vaccines were introduced to protect against *B. pertussis* infection (13). Widespread use of DTP in the United States led to a 90% decrease in the number of reported *B. pertussis* infections (13). Despite the efficacy of this vaccine, serious adverse side effects ensued, leading to the development of acellular pertussis vaccines. The antigens that are included in the current aP vaccines contain: filamentous hemagglutinin (FHA), fimbriae (FIM), PT, and pertactin (PRN). After the switch from the wP to the aP vaccines, there has been a significant increase in the number of pertussis cases in the United States and Europe (14). While nationwide vaccine coverage is 95% in the United States, the incidence of *B. pertussis* infections rose in the past 10 years as the number of aP-vaccinated-only population has increased in size. (15). While vaccine coverage remains high, the population dynamics are changing, and more people are aP immunized as the new generations are born and as the wP-only generations age. Numerous studies have demonstrated waning efficacy of aP vaccines in parallel with the emergence of genetically divergent strains of *B. pertussis* (3, 16–21). Increased surveillance of *B. pertussis* has led to the identification of clinical isolates that do not express PRN, FIM, and even PT, the hallmark toxin of the organism (22). One plausible hypothesis to explain these observations is that circulating *B. pertussis* strains have evolved due to acellular vaccine pressure. While we know the current strains are genetically different, we do not know whether this genetic variability affects the virulence, disease burden, toxicity, or fitness of the pathogen (3).

Early rat studies were performed with intranasal administration of *B. pertussis* for infection (6). Subsequent rat challenge studies used *B. pertussis* encased in agar beads for intrabronchial instillation (7–11, 23). While the agar bead infection method was successful at establishing infection, we aimed to utilize the simplicity of intranasal administration. We sought to reinvestigate the coughing rat model of pertussis to compare the pathogenesis of reference strain Tohama 1 to the recent clinical isolate D420, which has been extensively studied in the baboon model of pertussis (24–30). Tohama 1 was first isolated from a case of whooping cough in 1954, while D420 was isolated in 2002 from a critically ill infant in Texas (25, 31). Although Tohama 1 is now a reference strain that has been widely used since Sato and Sato developed the aP, but recent data have shown that this strain is an considered an outlier (32, 33). Tohama 1 does express PRN but has lower expression of PT and ACT (34). When Tohama 1 was used as a challenge strain in baboons, the baboons did not exhibit symptoms of pertussis despite being infected (26). This led to the selection of recent clinical isolate D420 as the baboon challenge strain, which readily infected and caused disease (26). With 48 years of potential genetic divergence, we sought to understand the differences in pathogenesis between these two commonly studied strains. D420 is known to infect mice and baboons, and it belongs to the clade (CDC013) of strains that represented 50% of isolates recovered in the United States in 2000 (3). However, it is important to note that D420 has an intact pertactin gene, and it does express the PRN protein, as confirmed by shotgun proteomic analysis (data not shown).

In the present study, we aimed to reestablish an upper respiratory tract infection model in rats following intranasal challenge with Tohama I and D420. We hypothesized that the recent isolate D420 would induce a more severe disease profile compared to Tohama 1, since previous research investigating pathogenesis in rhesus macaques noted that Tohama 1-infected animals did not exhibit overt disease symptoms (26). Sprague-Dawley rats were intranasally challenged with *B. pertussis*, and we characterized their disease progression profile over a 12-day infection. Cough was critically assessed utilizing whole-body plethysmography (WBP). Bacterial colonization, leukocytosis, and serological responses were measured as a result of infection. Rats challenged with D420 had increased coughing, greater bacterial burden in the respiratory tract, and a more robust IgM antibody response compared to rats challenged with Tohama 1. The coughing rat model of pertussis can shed light on the pathogenesis of *B. pertussis* and will likely be a useful tool for pertussis vaccine evaluation.

## RESULTS

### D420-infected rats have increased number of coughs compared to strain Tohama 1 over the entire course of infection.

**FIG 1:**
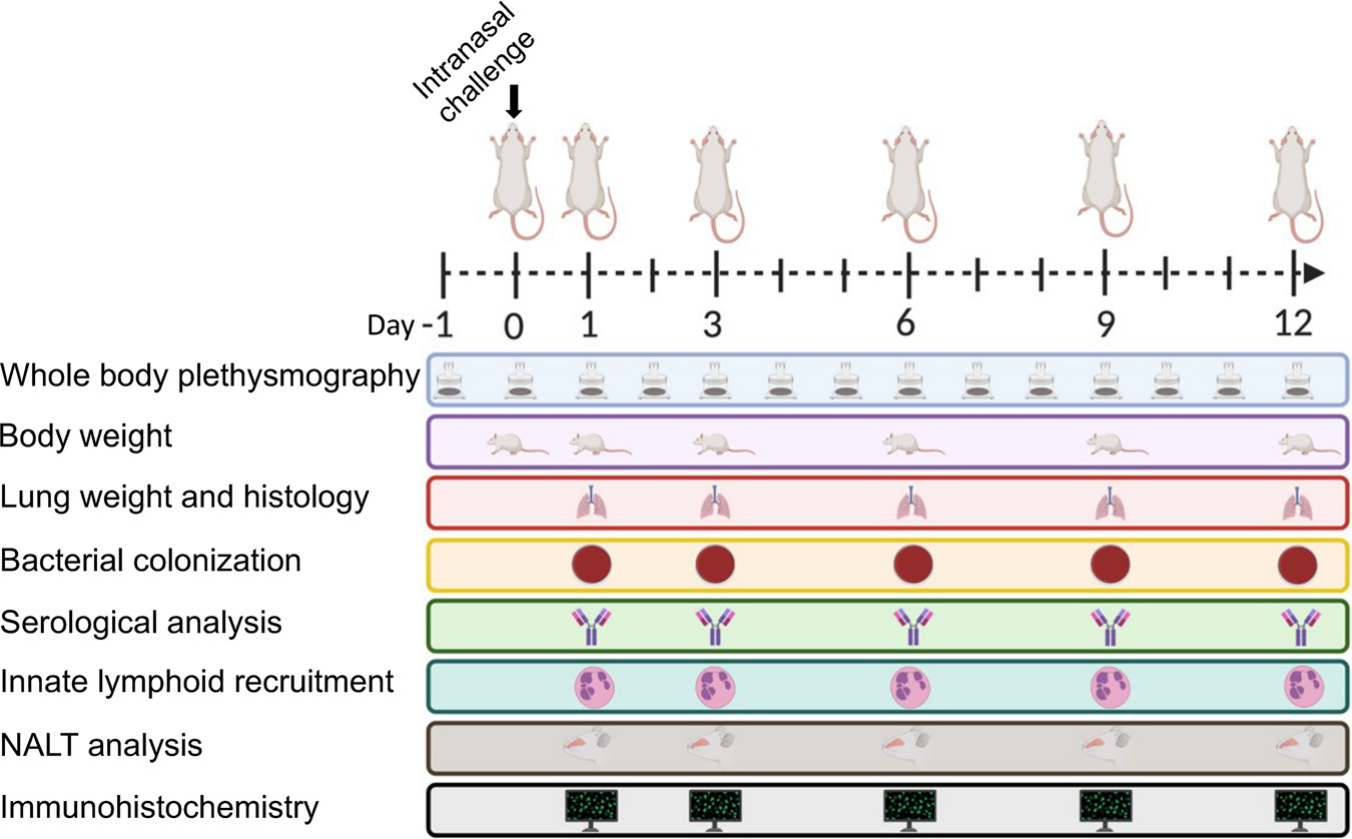
Experimental design of intranasal infection of *B. pertussis*. Schematic representation of Sprague-Dawley rats intranasally infected with *B. pertussis*. Whole-body plethysmography was used to measure cough and analyze lung function over the course of a 12-day infection. Body weight, bacterial burden of the respiratory tract, innate lymphoid recruitment, and antibody titers were measured at the days 1, 3, 6, 9, and 12 postchallenge. The left lobe of the lung and the NALT were sectioned and stained with H&E for histological analysis, and immunohistochemistry was performed to visualize the bacteria in the left lobe of the lung and nasal cavity. (Figure 1 was created with BioRender.com.)

**FIG 2:**
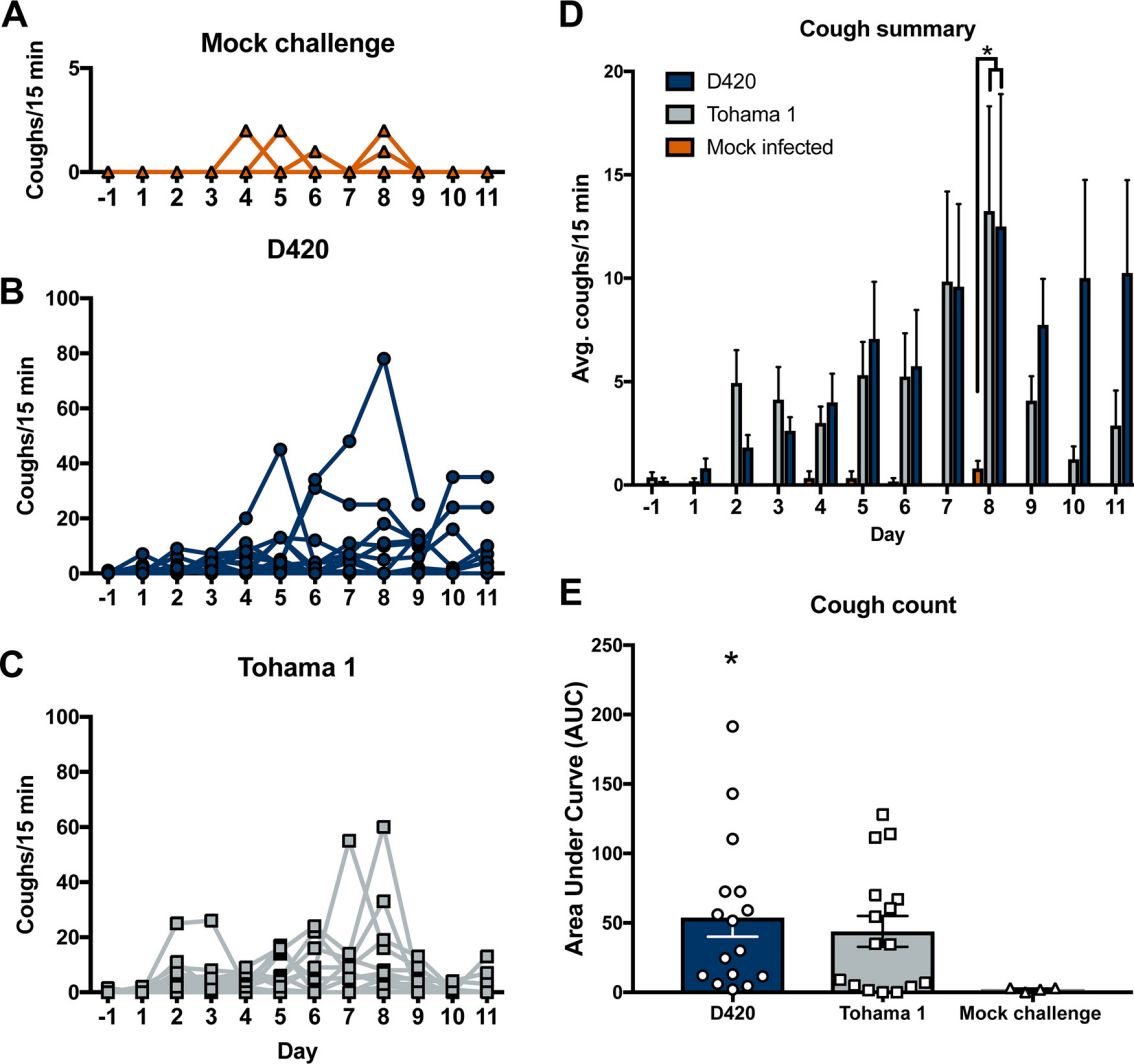
Infection of B. pertussis induces cough in Sprague-Dawley rats. Coughs were measured each day of the 12-day infection using whole-body plethysmography. Coughs were counted for mock challenge rats (A), rats infected with D420 (B), and rats infected with Tohama 1 (C). (D) Summary of results (see panels A to C) shown as means ± the standard errors of the mean (SEM; *n *= 4 to 8). (E) AUC, represented as averages of coughs per 15 min for each rat. *P* values were determined by two-way ANOVA, followed by a Bonferroni comparison test and an unpaired Student *t* test for AUC (*, *P < *0.05 [D420 compared to the mock-challenged control group], #, *P* < 0.05 [Tohama 1 compared to the mock challenged control group]).

Over the past decade it has become clear that improvements to the acellular pertussis vaccine strategy are needed due to the increases in PRN mutants, genomic divergence, and epidemiological data. We believe that in order to improve pertussis vaccines we need to better understand the pathogenesis of *B. pertussis* in an animal model that shares similar clinical manifestations of pertussis seen in humans. Here, we intranasally infected 7-week-old Sprague-Dawley rats with 10^8^ CFU of *B. pertussis* strain D420 or Tohama 1 (Fig. 1) in an effort establish infection and observe *B. pertussis* induced cough. For negative controls (no bacterial challenge), rats were intranasally administered sterile phosphate-buffered saline (PBS). Paroxysmal cough is the hallmark symptom of pertussis and is thought to play a major role in transmission of the organism to a new host. To quantify respiratory function during infection, we utilized whole-body plethysmography (WBP). WBP instrumentation consists of specialized containment chambers which monitor box flow, temperature, and airflow changes to measure respiratory function. Counting coughs via WBP provides an extremely accurate and unbiased way of counting coughs based on cough waveforms. Rat containment chambers were placed inside a laminar flow hood and connected to a computer that would monitor the rats breathing (see Fig. S1). Early studies reported that *B. pertussis* infection in young rats induced coughs that were audible by ear (6), and in our preliminary studies this was apparent to us as well (data not shown). Studies in the 1990s of paroxysmal cough in rats were quantified using an analog sound recording device (7). We hypothesized that rats infected with the recent isolate D420 would induce more coughs than animals infected with the reference strain Tohama 1 because rhesus macaques infected with Tohama 1 did not exhibit overt disease symptoms but D420 induced robust coughing in baboons (26). *B. pertussis*-infected rats developed cough at days 2 to 11 (Fig. 2B and C), while mock-infected rats (Fig. 2A) only had a few isolated coughs, unrelated to infection. At days 1 to 3 postinfection, the average cough count for rats infected with Tohama 1 or D420 was fewer than 5 coughs per 15 min. The average cough count doubled by day 7 postinfection, with peak coughing occurring at day 8 postinfection and with an average of 13 coughs per 15 min of monitoring. After day 8, the average cough count for rats infected with D420 remained above 10 coughs per 15 min, while the rats infected with Tohama 1 averaged fewer than 5 coughs. To summarize the cough data, the average cough count over the course of infection is shown (Fig. 2D). We observed a significant increase in the number of coughs at day 8 postinfection of *B. pertussis*-infected rats compared to the mock-infected challenge control. Rats infected with D420 coughed a total of 949 times, whereas rats infected with Tohama 1 coughed a total of 724 times over the entire 12-day infection. To analyze differences in cough count between strains, we utilized area under the curve (AUC) analysis (Fig. 2E). AUC analysis allows us to quantify the number of coughs over the entire course of infection per rat. Using this analysis approach, we note a significant increase in the number of coughs for rats infected with D420 compared to the mock-challenged rats over the 12-day infection, while there was no significant difference observed in Tohama 1-infected rats compared to mock-challenged rats (noninfected).

### *B. pertussis* infection causes pulmonary distress.

**FIG 3:**
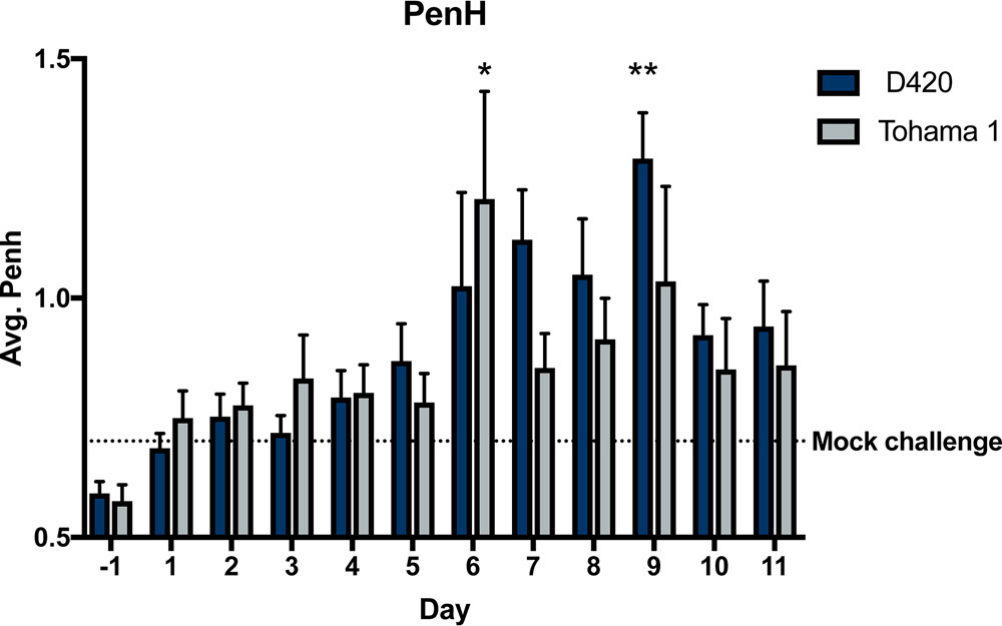
B. pertussis infection impairs respiratory capacity. Over the course of infection, Sprague-Dawley rat respiratory function was analyzed using whole-body plethysmography. Each day at 5 p.m., rat bronchial restriction was determined utilizing WBP to calculate the PenH. The results are shown as means ± the SEM (*n* = 4 to 8). *P* values were determined by two-way ANOVA, followed by Dunnett’s comparison test (*, *P < *0.05; **, *P < *0.01 [compared to the mock challenge group]).

Infection with *B. pertussis* leads to mucus production, lung damage, and invasion of cellular infiltrates into the bronchioles of the lung (35). The lungs of infants with *B. pertussis* exhibit edema, necrotizing bronchiolitis, and inflammation in the lung leading to respiratory distress (35). WBP was used to quantify the pulmonary distress over the course of infection. Pulmonary distress was measured by calculating the enhanced pause (PenH) of the animal (Fig. 3). The higher the PenH value, the increased respiratory distress of the animal. PenH corresponds to the resistance associated with peak inspiratory height and peak expiratory height and taking into account time between early and late expiration per breath (see Fig. S2). With increased constriction, the expiratory peak becomes prominent and the area of the peak expiratory height becomes a larger percentage of the complete expiratory area. Other factors that contribute to the increased PenH is a decrease in relaxation time (Tr) in relation to expiratory time (Te). Previous research has shown that severe inflammation in the lungs of mice directly correlated with an increase in PenH (36). More recently, SARS-CoV-2 infected mice were observed to have subtle but significant increase in PenH postchallenge (37, 38). Compared to the mock-challenged rats, both D420- and Tohama 1-infected rats displayed an increase in PenH (Fig. 3). We observed a significant increase at day 9 with rats infected with D420 and at day 6 with rats infected with Tohama 1 compared to mock-challenged rats.

### Histological assessment of the lung and nasal cavity.

**FIG 4:**
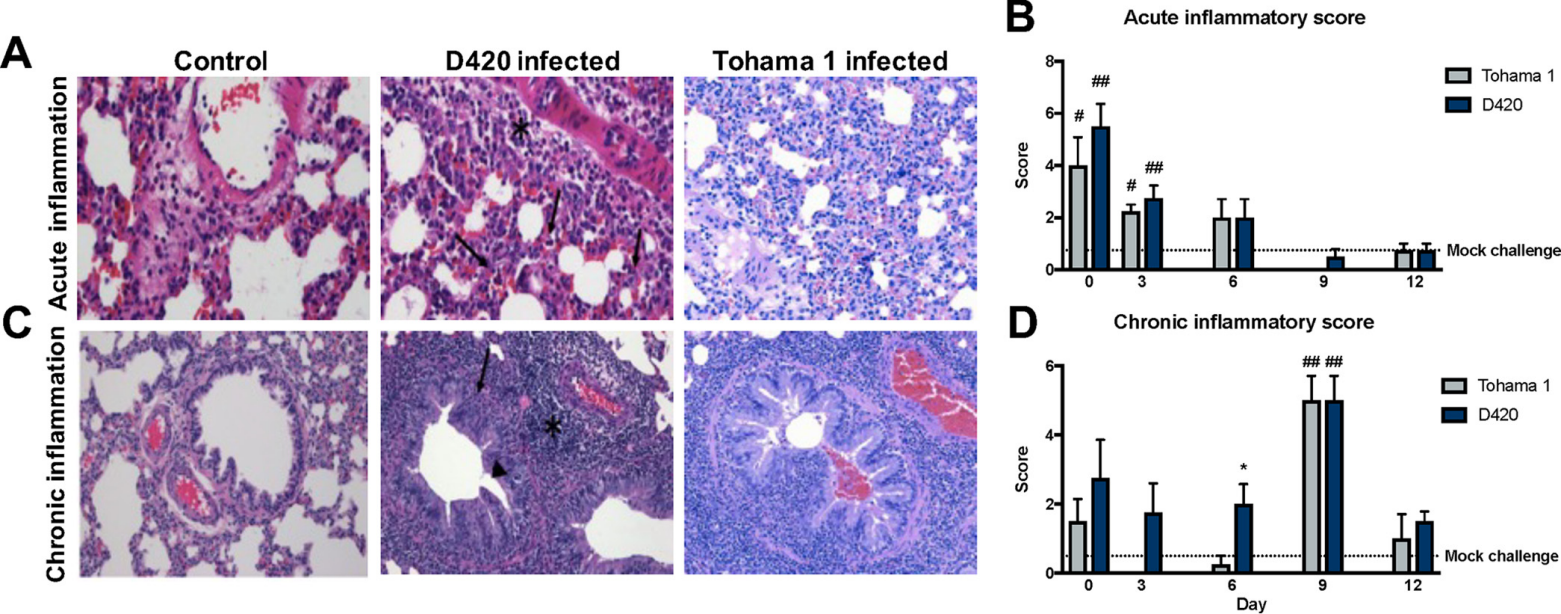
*B. pertussis* infection induces acute and chronic inflammation in the lungs of Sprague-Dawley rats. The left lobe of the lung was sectioned and stained with H&E from rats infected with D420 or Tohama 1 or from the PBS control. (A) Representative images of acute inflammation demonstrating neutrophil recruitment around surrounding blood vessel (asterisk) and parenchyma (arrows) at ×400 magnification. (B) Average acute inflammatory score of the lung based on the predominance of neutrophils in the parenchyma, blood vessels, and airways. (C) Representative images of chronic inflammation showing mononuclear cells surrounding the blood vessel (asterisk), lamina propria (arrow), and bronchioles (arrowhead) at ×200 magnification. (D) Average chronic inflammatory score of the lungs characterized by mononuclear infiltrates in the parenchyma, blood vessels, and airway. Histological assessment was determined blinded with no knowledge of the treatment groups. The results are shown as means ± the SEM (*n* = 4). *P* values were determined by one-way ANOVA, followed by Dunnett’s comparison test (*, *P < *0.05 [compared between challenge groups]; #, *P < *0.05, ##, *P < *0.01 [compared to mock challenge]).

**FIG 5:**
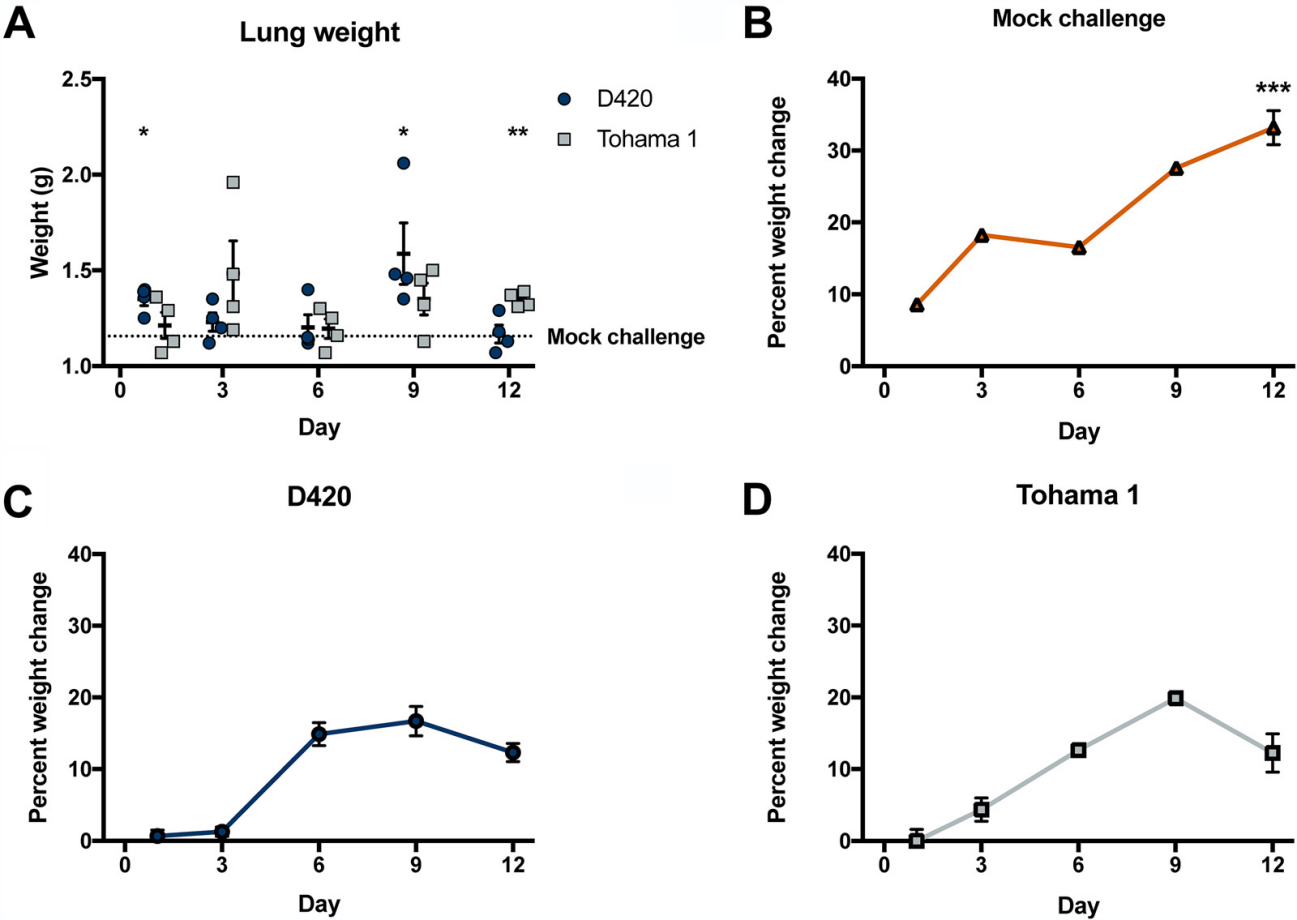
*B. pertussis* infection induces inflammation of the lung and a decrease in weight gain. (A) After euthanasia, the lung weight was measured and recorded. To observe any weight changes over the course of infection, we calculated the percent weight change for mock-challenged (B), D420-infected (C), and Tohama 1-infected (D) rats. Rat body weights were recorded before infection, as well as immediately posteuthanasia. The percent weight change was calculated by taking the differences between starting weight and end weight and dividing by the initial weight and then multiplying by 100. Results are shown as means ± the SEM (*n* = 1 to 4). Only one mock-challenge rat weight was measured at days 1, 3, 6, and 9. Weights of 4 mock-challenge rats were recorded at day 12 postinfection. *P* values were determined by one-way ANOVA (*, *P* < 0.05; **, *P* < 0.01; ***, *P* < 0.001 [(A) compared to mock-challenge group; (B to D) compared to both infection groups]).

To further evaluate the inflammation in the lung, we utilized histology to confirm both acute and chronic inflammation in response to *B. pertussis* infection. At days 1, 3, 6, 9, and 12 postchallenge, the left lobes were collected, sectioned, and stained with hematoxylin and eosin (H&E) (Fig. 4A and C). Whole-lung images were taken to assess cellular infiltration (see Fig. S3). Once stained, the blinded slides were then scored by a board-certified pathologist. Mock-challenged animals exhibited minimal inflammatory infiltrates consisting of focal accumulations of mononuclear cells in the parenchyma and occasional infiltrates of neutrophils surrounding blood vessels (Fig. 4A). Rats infected with Tohama 1 and D420 showed significant increases in their acute inflammation scores compared to mock-challenged animals at days 1 and 3 postinfection (Fig. 4B). The highest acute inflammation score occurred at day 1 postinfection (Fig. 4B). Rats exhibited mild to moderate neutrophil infiltration of the parenchyma, blood vessels, and bronchioles (Fig. 4A). Markers of acute inflammation resolved after day 6 postchallenge; however, infected rats at day 9 exhibited obvious differences in the lungs associated with mild to moderate infiltration of mononuclear cells and higher chronic inflammation compared to mock-challenged animals (Fig. 4C). At day 12 postinfection, we notice moderate resolution of inflammation in both D420- and Tohama 1-infected rats (Fig. 4B and D). We also observed a significant increase in lung weight, which can be associated with elevated inflammation, at days 1 and 9 postchallenge in D420-infected rats compared to the mock-challenged controls (Fig. 5A). At day 12 postchallenge, rats infected with Tohama 1 showed a significant increase in lung weight compared to the mock-challenged rats (Fig. 5A). These results confirm that *B. pertussis* infection induces inflammation in the lungs of coughing Sprague-Dawley rats.

**FIG 6:**
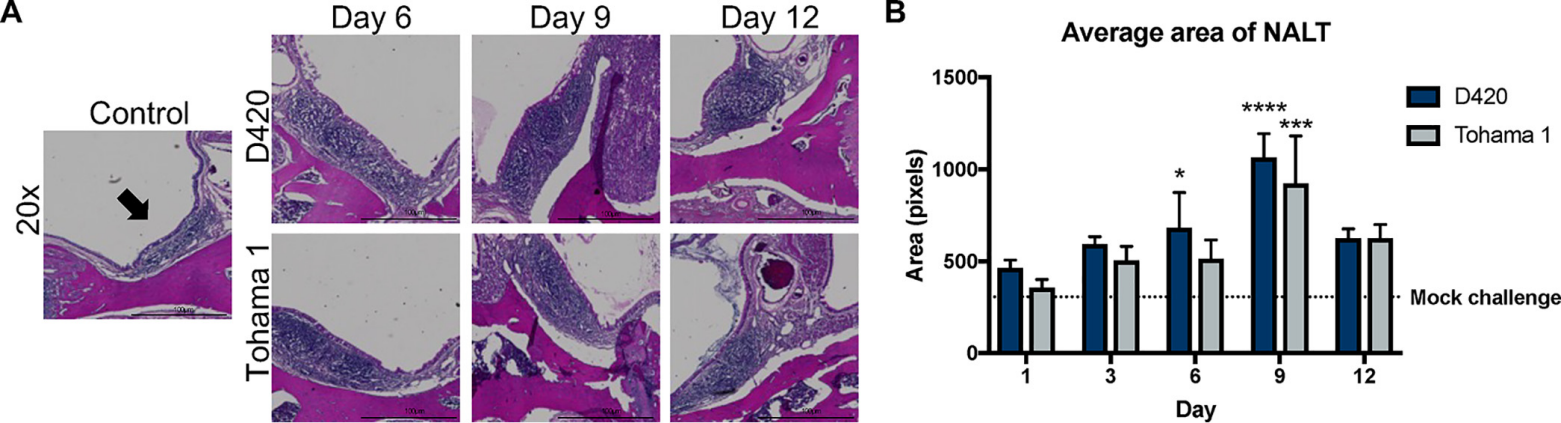
Analysis of the nasal associated lymphoid tissue (NALT). The skull was sectioned, allowing staining of the NALT with H&E. (A) Representative 20× images of the NALT. Arrows indicate the NALT. (B) ImageJ analysis of the area of both left and right NALT. The dotted line represents the average area of the NALT in the mock-challenged group. Results are shown as means ± the SEM (*n *=* *4). *P* values were determined by two-way ANOVA (*, *P < *0.05; ***, *P < *0.001; ****, *P < *0.0001 [compared to the mock-challenge group]).

Due to the fact that *B. pertussis* is a mucosal pathogen, we sought out to investigate any phenotypic changes or inflammation in the nasal-associated lymphoid tissues (NALT) of *B. pertussis*-infected rats via histology. NALT helps elicit immunity against airborne and mucosal pathogens (39). The NALT is located above the hard palate, and studies have also shown that with the introduction of antigen via vaccination or bacterial components, cellular expansion of the NALT ensues (40–44). The NALT was stained with H&E, and we observed an increase in cellular infiltrate and a size enlargement in the NALT of *B. pertussis*-infected rats compared to the mock-challenged rats (Fig. 6A). ImageJ analysis was used to measure the area of both the left and right NALT of each animal (Fig. 6B). These data could mark the potential cellular expansion of the NALT upon infection with *B. pertussis*. Further analysis of the expanding cell populations in the NALT will warrant more analysis especially in the context of vaccine immunity.

### D420-infected rats demonstrate an increased bacterial burden in the respiratory tract.

**FIG 7:**
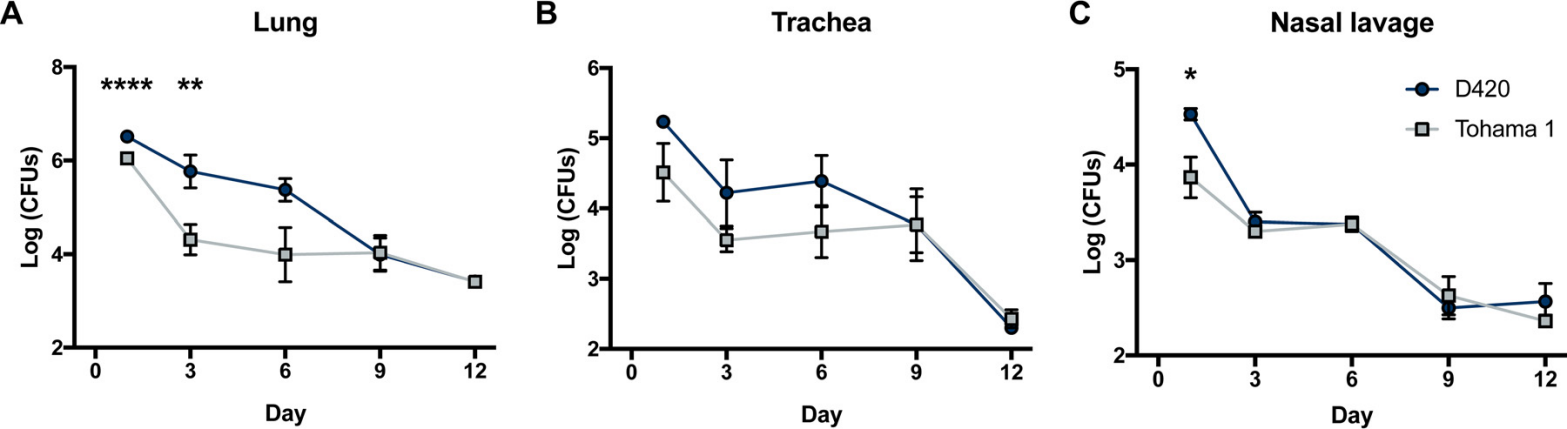
Analysis of bacterial burden over the course of *B. pertussis* infection. Bacteria were quantified by serially diluted CFU following intranasal challenge. CFU counts were determined from lung homogenate (A), trachea (B), and nasal lavage (C). The results are shown as means ± the SEM (*n *=* *4). *P* values were determined by two-way ANOVA corrected with the Bonferroni comparison test (*, *P < *0.05; **, *P < *0.01; ****, *P < *0.0001 [compared between D420 and Tohama 1]).

We determined the bacterial burden in the respiratory tract for Tohama I- and D420-challenged rats at days 1, 3, 6, 9, and 12. Over the coughing timeline, we observed an overall decrease in viable bacteria in the lung, trachea, and nasal cavity. In the lung, we observed a significant increase in the recovered bacteria in D420-infected rats compared to Tohama 1-infected rats at both days 1 and 3 (Fig. 7A). The same trend is also seen in the nasal cavity at day 1 postinfection (Fig. 7C). We observed no difference in the viable bacteria recovered from the trachea (Fig. 7B). Previous pertussis studies utilizing the rat model of pertussis noted weight loss from intrabronchial infection of *B. pertussis* strain 18-323 (10). *B. pertussis* infection is sufficient to cause weight loss in rats (10). To confirm this using intranasal infection, body weights were measured before challenge and upon euthanasia to calculate the percent weight change. We observed a significant decrease in weight gain in rats infected with *B. pertussis* strain D420 and Tohama 1 compared to the mock-challenged animals at day 12 postinfection (Fig. 5B to D).

Next, we aimed to compare the bacterial colonization between outbred mice and rats in an effort to relatively compare the bacterial burden between models. We utilized the bacterial burden data in CD1 mice and compared them to our data in rats (45). To address the body weight differences between the mice and rats, we used the CFU/organ and divided these values by their respective body weights for a crude comparison. Our data suggest that despite having a higher initial challenge dose in the rat, the bacterial burden remaining in the respiratory tract is higher in a mouse compared to a rat later during infection (see Fig. S4). In general, the rat model is likely a lower challenge dose per body weight model of pertussis compared to mouse models.

### Immunofluorescence confirms colonization of *B. pertussis* in the lungs and nasal cavity.

**FIG 8:**
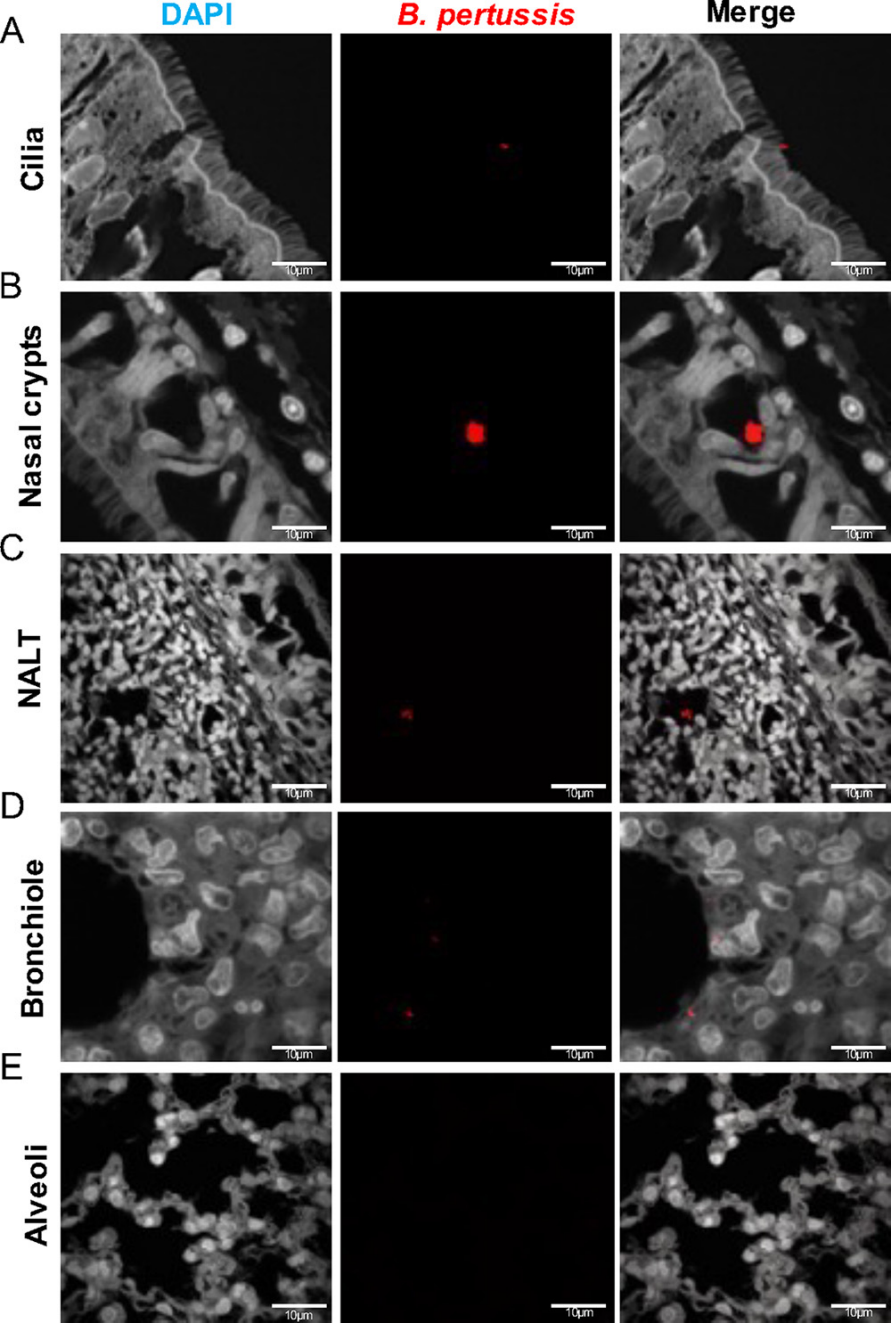
Immunofluorescence (IF) staining of *B. pertussis* localization in the respiratory tract. *B. pertussis* was labeled using a polyclonal antibody to FHA and counter-tagged with a fluorescently conjugated antibody (Texas Red). Sections were counterstained with DAPI. (A to C) Representative images of *B. pertussis* in the nasal cavity over the course of infection. *B. pertussis* was found captured in the cilia of the nasal cavity, as well as of the NALT. (D and E) Representative images of the bronchioles and alveoli of infected rats. *B. pertussis* was found localized in the bronchioles over the course of infection and absent in the alveoli.

We next wanted to assess the location of bacterial colonization in the nasal cavity and visualize any bacteria left behind following flushing of the nares. We sectioned the skulls after flushing the nasal cavity and utilized immunofluorescence (IF). After visually scanning the nasal cavity using confocal microscopy, we found bacteria in the nasal turbinates and the NALT (Fig. 8A and C). We also confirmed that the bacteria were found between the epithelial cells (Fig. 8B). The relative locations where the bacteria were found in the nasal cavity can be seen in Fig. S5A in the supplemental material. Our results show that *B. pertussis* remained in the nasal cavity over the entire course of infection even after flushing the nares (see Fig. S5B). The flushing of the nasal cavity underrepresents the total number of *B. pertussis* in the nasal cavity. In the lung, *B. pertussis* colonizes the bronchioles, while the alveolar spaces remain noncolonized (Fig. 8D and F). IF was performed on the nasal cavities and lungs of mock-challenged animals to access nonspecific binding (see Fig. S6). To assess any potential differences between the strains we blindly manually counted the labeled microcolonies in the lung and nasal cavity. It is also worthy to note, we found no differences in microcolony counts in the lung or nasal cavity between the strains (see Fig. S5C and D) despite our observed differences in bacterial burden by CFU enumeration. CFU is likely a better indicator of overall bacterial burden, but IF revealed that some *B. pertussis* remains in the airway following flushing of the nares.

### Measurement of recruitment of neutrophils in response to *B. pertussis* infection.

**FIG 9:**
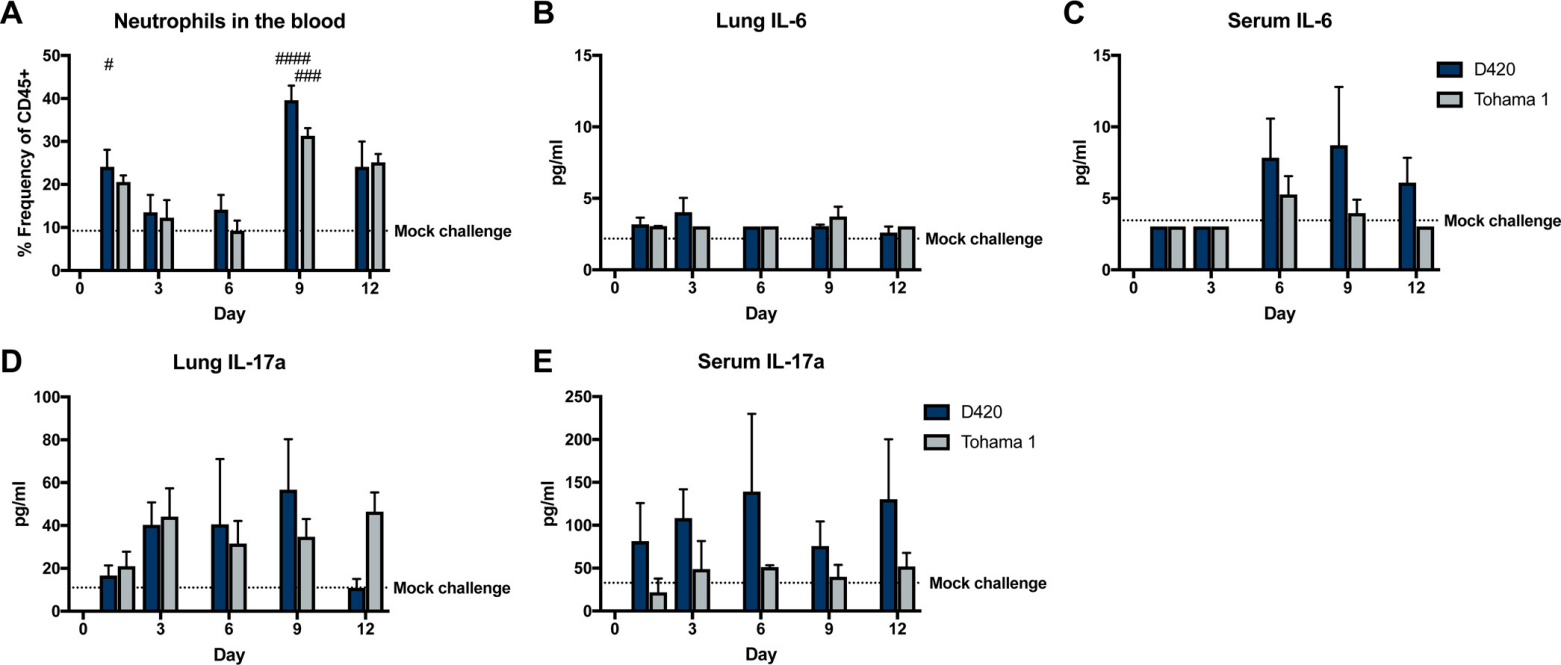
Neutrophil recruitment and proinflammatory cytokine production after challenge determined by flow cytometry and multiplex immunoassay analysis. (A) Blood neutrophil count of Sprague-Dawley rats infected with Bordetella pertussis. At days 1, 3, 6, 9, and 12 postinfection, rats were euthanized, and blood was collected via cardiac puncture. Flow cytometry was used to quantify the amount of neutrophils (CD45^+^ CD161^−^ B220^–^ CD43^+^ His48^hi^) in the blood. Quantification of the percentage of single, CD45^+^ cells was performed. The dotted line represents the average frequency of CD45^+^ neutrophils in the mock challenge animals over the course of the study. (B and C) Analysis of cytokine IL-6 from the supernatant of lung homogenate and serum. (D and E) Analysis of cytokine IL-17 in serum and lung homogenate supernatant. Cytokines were analyzed using a ProcartaPlex multiplex immunoassay kit. Results are shown as means ± the SEM (*n *=* *4). The dotted line represents the average mock-challenged cytokine levels measured. *P* values were determined by one-way ANOVA, followed by a Tukey comparison test (#, *P* < 0.05; ##, *P* < 0.01; ####, *P* < 0.0001 [compared to mock challenge]).

Previous studies utilizing the rat model of pertussis noted leukocytosis and an increase in the number of total white blood cells when agar-encased *B. pertussis* was used for infection (8). Neutrophils circulating through the blood were measured during intranasal infection by antibody staining and flow cytometry, since *B. pertussis* infection results in various amounts of neutrophilia (35, 46). One caveat in analyzing cell populations via flow cytometry in the rats is the limited availability of rat specific antibodies compared to mice. Recent research by Barnett-Vanes et al. has developed a flow cytometry panel to analyze cell populations in a model of lipopolysaccharide-induced pulmonary inflammation (47). The percentages of CD45^+^ (CD161^−^ B220^−^ CD43^+^ His48^Hi^) neutrophils in the blood were assessed in *B. pertussis*-infected rats compared to mock-challenged animals (Fig. 9A). Specifically, a significant increase was observed in the number of circulating neutrophils in the blood of rats infected with D420 at days 1 and 9, with Tohama 1-infected rats only having a significant increase in response to mock challenge at day 9 (Fig. 9A). Next, cytokine concentrations in the lung and serum were measured to identify factors that could contribute to the recruitment of neutrophils, specifically interleukin-6 (IL-6) and IL-17. IL-6 is a potent inducer of Th17 polarization and has been found elevated during murine infection with *B. pertussis* (45, 48–50). Surprisingly, we did not observe any significant changes in IL-6 due to *B. pertussis* infection (Fig. 9B and C). It has been well documented in the mouse model that the production of IL-17 in pertussis plays a role in the increase in circulating neutrophils (51). There were no significant changes in IL-17a in the sera or lungs of *B. pertussis*-infected rats compared to mock-challenged rats (Fig. 9D and E). Th1 and Th2 cytokines and chemokines in the lung and serum were also measured (see Fig. S7 to S10). We did not observe an increase in the induction of cytokines by infection overall; however, in the serum, rats infected with D420 had a significant increase in IL-5 compared to Tohama 1-infected rats at day 12 postinfection (see Fig. S7). There was a significant increase in IL-5 and TNF-α at day 6 postinfection in the lung in rats infected with Tohama 1 compared to D420 (see Fig. S8). We also observed significant increases in chemokines MIP-1a, RANTES, MCP-3, MIP-2, and IP10 in rats infected with D420 at day 12 postchallenge in the serum compared to rats infected with Tohama 1 (see Fig. S19). In the lung, MIP-2 and IP10 were increased in rats infected with D420 compared to the mock-challenged controls (see Fig. S10). Despite slight differences highlighted above we did not observe a massive difference in the cytokine and chemokine responses during infection.

### Serological analysis of *B. pertussis*-specific antibodies.

**FIG 10:**
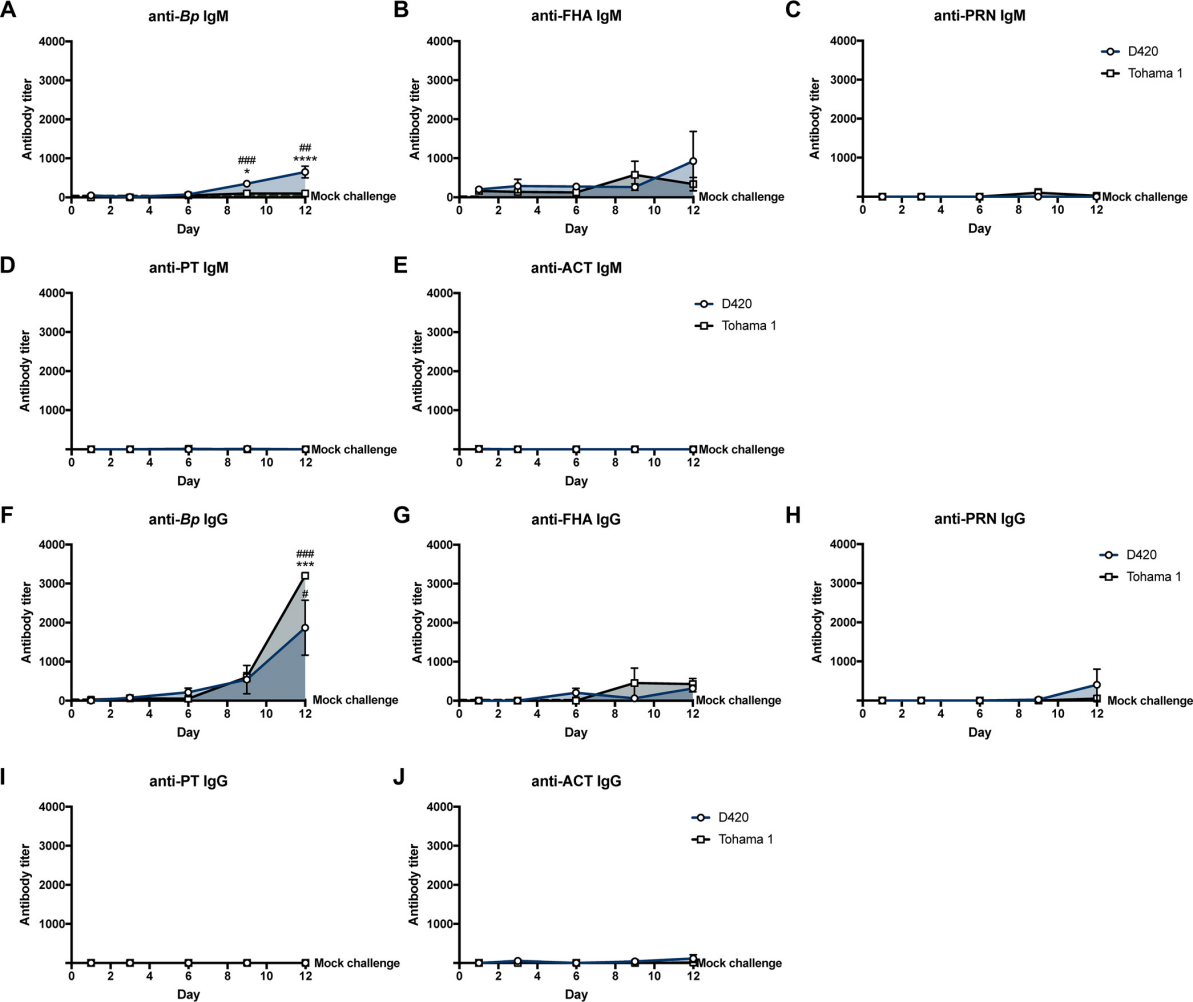
Measurement of serum antibody titers over the course of B. pertussis infection. ELISA was used to compare serological responses from rats intranasal challenge with B. pertussis. Total IgM and IgG serum antibody titers were measured from challenged rats against whole B. pertussis bacterium (A and F), FHA (B and G), PRN (C and H), PT (D and I), and ACT (E and J). The dotted line represents the average serum titer for mock-challenged rats. *P* values were determined by two-way ANOVA corrected with Bonferroni comparison test (*, *P < *0.05; ***, *P < *0.001; ****, *P < *0.0001 [compared between infection groups]). For comparison to the mock-challenged control group, one-way ANOVA was used, followed by a Tukey comparison test (##, *P* < 0.01; ###, *P* < 0.001).

Enzyme-linked immunosorbent assays (ELISAs) were performed to determine the serological response generated from *B. pertussis* infection. We measured IgM and IgG antibody titers in the serum to whole bacterium and *B. pertussis*-associated virulence factors at days 1, 3, 6, 9, and 12 postinfection. In previous studies utilizing the rat model, intrabronchial infection induced the generation of IgG antibody titers to sonicated *B. pertussis* at 28 days postinfection (8). Intranasal infection of strain D420 has generated significant anti-PT IgG antibody titers in the baboon model at days 17 to 19 postinfection (26). However, analysis of antibody titers generated against an intranasal pertussis infection in the rat has yet to be determined, and we wanted to characterize the primary antibody response to *B. pertussis* (see Fig. S11). We observed a significant 10-fold increase in the anti-*B. pertussis* IgM antibody response at day 9 and an almost 20-fold increase 12 days postchallenge in rats infected with D420 compared to the mock challenge, while we also saw a significant 6-fold increase in anti-*B. pertussis* IgM at day 12 postinfection compared to the amount of anti-*B. pertussis* IgM from Tohama 1 infection (Fig. 10A). At day 12 postinfection, we observed that the *B. pertussis* infection induced significant production of anti-*B. pertussis* IgG in the serum compared to mock-challenged animals (Fig. 10F). At day 12 postinfection, Tohama 1-infected rats showed a significant increase, nearly double anti-*B. pertussis* IgG antibody titers compared to rats infected with D420. AUC analysis revealed a significant increase in anti-*B. pertussis* IgM antibodies from rats infected with D420 compared to Tohama 1-infected rats (see Fig. S12A). However, no difference was observed in the AUC values of the anti-*B. pertussis* IgG antibody response over the total course of infection between the bacterial challenge groups (see Fig. S12B). It is also important to note that we also did not see a significant increase in the generation of antibodies titers to PT, ACT, PRN, and FHA in *B. pertussis*-infected rats compared to mock-challenged animals (Fig. 10B, E, and G to J). We also note that IgA antibodies were not detected to either whole bacteria or PT in the lung and nasal cavity at days 1 and 9 postchallenge (data not shown).

## DISCUSSION

To date, humans are the only known reservoir for *B. pertussis*, making the development of a suitable animal model challenging. Multiple models have been studied to recapitulate similar disease symptoms seen in humans, as well as provide insight into the pathogenesis of *B. pertussis*. We set out to reinvestigate the rat model of pertussis that was originally introduced in 1938 (6). In our study, we have confirmed that intranasal administration of *B. pertussis* led to bacterial colonization of the upper and lower respiratory tracts (Fig. 7). Through intranasal instillation, bacteria were able to infect the nasal cavity, trachea, and lungs postchallenge. This is important since infants with pertussis experience pneumonia more frequently than do adolescents and adults (35). Mouse models of pertussis also rely on depositing large numbers of bacteria into the lung to establish infection (2). Here, our data show that through intranasal infection, we can establish an infection of both the upper and lower respiratory tracts, resembling the infection seen in adolescents and adults with pertussis (52, 53).

Previous reports have shown *B. pertussis* infection of rats induced paroxysmal coughs, and these coughs were counted by analog audio recordings (7). Utilizing WBP, we are able to precisely and digitally count coughs and measure the respiratory capacity for the first time with rats infected with *B. pertussis* (Fig. 2 and 3). Here, coughs induced from infection appeared at days 1 to 2 postchallenge, with the number of coughs gradually increasing with peak cough occurring at day 8 postchallenge. Rats challenged with intrabronchial instillation of *B. pertussis* noted similar peak cough counts approximately 10 days postchallenge (54). This is also one of the first studies utilizing WBP to count coughs in rats with bacterial infection. We did observe some infected rats that did not overtly cough. This reflects the frequency of coughs in adolescents and adults (55). Previous studies using baboons also show that *B. pertussis* infection elicits severe coughs lasting over 2 weeks postchallenge, with peak coughs per hour occurring at day 4 postchallenge (26). It is also important to note that at peak coughing, the bacterial burden was low, which was also observed in previous research on *B. pertussis*-infected rats (7). The data suggest that other factors could be playing a role in cough production despite the bacterial infection being resolved. Given that *B. pertussis*-infected rats coughed, we also investigated transmission of *B. pertussis* from infected 7-week-old rats (actively coughing) cohoused with more susceptible 3-week-old rats, and we have observed no evidence of transmission via negative qPCR (IS*481*) analysis of swabs from the nasal cavity and no active coughing in the naive 3-week-old rats. We also performed a challenge study with *B. bronchiseptica* strain KM22 in 7-week-old Sprague-Dawley rats, and challenged rats coughed more violently and earlier after bacterial challenge compared to *B. pertussis*-infected rats. *B. bronchiseptica*-infected rats coughed the most when the bacterial loads in the respiratory tract were at their highest, which could potentially lead to transmission of the bacterium. TLR4-deficient rats could be investigated to evaluate transmission of *B. pertussis* (56). We also observed that the increased bronchial restriction coincides with the increased coughs during days 6 to 11 postchallenge (Fig. 3). The decreased lung capacity and increase in cough could be further associated with an increase of chronic inflammation in the lung (Fig. 4). Our data suggest that the coughing rat model of pertussis can be used to potentially evaluate the mechanism of cough, which still requires further investigation.

Initial studies characterizing the serum antibody response to rats infected with *B. pertussis* noted an increase antibody response to endotoxin correlated with the small number of cultivable organisms in the lung following intrabronchial challenge (7). Hall et al. demonstrated that rats infected with *B. pertussis* become seropositive in response to whole-cell *B. pertussis* lysate, FHA, and PT by 28 days after intrabronchial challenge (8). Anti-FHA antibody titers were not detected until day 16 postchallenge, whereas anti-PT antibody titers were not detected until day 28 postchallenge (23). In our study, we characterized the antibody response following intranasal challenge of *B. pertussis* over the whole infection timeline. Using *B. pertussis*-coated ELISA plates, we detected anti-*B. pertussis* IgG antibody titers in rats infected with *B. pertussis* at day 9 postchallenge, with a significant increase compared to mock challenge observed at day 12 (Fig. 10). We also measured a significant increase in anti-*B. pertussis* IgM antibody titers in rats infected with D420 compared to the mock-challenged and Tohama 1-infected animals (Fig. 10). One potential hypothesis that could explain this observation is the genetic differences between the two strains as D420 harbors the *ptxP3* allele, leading to an increase in PT production (25). PT has been shown to suppress *B. pertussis* antibody responses in serum and limit the expression of antigen-presenting receptors (57–60).

With the increased interest in developing new pertussis vaccines, it is imperative for the development of animal models to evaluate the pathogenic potential of current emerging strains, as well as to identify any potential differences in disease burden between genetically divergent strains. Sato and Sato first used *B. pertussis* strain Tohama 1 in the development of the acellular pertussis vaccine, and Tohama 1 is still being used in pertussis research today, notably due to its genetic malleability (61). D420 has been instrumental in the development of the baboon model and has also been used to study vaccine efficacy in regard to transmission in the baboon model (29). We hypothesize that the observed differences could be due to the increased amount of PT through the *ptxP3* allele of D420 (25). Strains, such as D420, harboring the *ptxP3* allele lead to an increase in virulence, PT production, and prevalence (62, 63). A previous study utilizing the intranasal mouse model has shown an increase in bacterial colonization with *ptxP3* strains (64). Parton et al. illustrated that infection with a PT-deficient strain was unable to induce cough (9). It is possible that there are other factors beyond PT that can account for the observed virulence differences between Tohama I and D420. In the rat model, unlike the mouse, we also did not measure any significant increases in IL-17 and IL-6 in the lung and serum after challenge. IL-17 and IL-6 are increased following *B. pertussis* infection in mice and baboons (30, 48, 65). Further investigation is needed to explain the observed differences. One potential approach to evaluate differences between strains is the use of a neonatal rat model of pertussis. Studying *B. pertussis* in neonates may be useful in distinguishing subtle differences between strains. Hornibrook et al. demonstrated that *B. pertussis*-infected young 3-week-old rats demonstrated 65% mortality, while 7-week-old rats do not succumb to infection with a similar dose (6).

In summary, we have demonstrated that the rat model of pertussis can be used as a tool to further study *B. pertussis* pathogenesis and recapitulate some of the similar symptoms of pertussis as seen in humans. Pertussis is often described as a “100-day cough” because adolescents and adults experience an extended duration of coughing (66). Coughing episodes in infants with pertussis can lead to vomiting, choking, gagging, and apneic episodes that can lead to seizures (67). Mice have not been used to evaluate coughing manifestation in pertussis until recently, and baboons are expensive and limited to specialized facilities (68). Use of the coughing rat model of pertussis provides feasibility through low husbandry costs, the ready availability of animals, and its ease of use as a model. The immunological tools available in murine research is vast, but as basic research continue to evolve, so, too, will the resources and immunological tools available for rats. Recently, flow cytometry markers have been developed to study innate immune cells and adaptive immune cells, such as B and T cells (47). In other studies, we have used ELISpot assays to investigate antigen-specific immune populations as well. The increasing availability of antibodies has also allowed investigation of systemic and mucosal serological responses. Future studies utilizing the coughing rat model of pertussis will capitalize on the growing immunological toolbox in regard to vaccination against pertussis.

In this study, we carefully compared two established strains that have been used in the pertussis field, Tohama 1 and D420. In future studies, we aim to evaluate whole-cell and acellular vaccine-mediated immunity against D420. Armed with this model, we can further our understanding of pathogenesis, host response during pertussis, genetic divergence between strains, and vaccine-mediated immunity.

## MATERIALS AND METHODS

### *Bordetella pertussis* strains and growth conditions.

*B. pertussis* strain Tohama 1 was graciously provided by Peter Sebo (Czech Academy of Sciences), and strain D420 was acquired from the CDC Pertussis Lab provided by Maria L. Tondella and Michael Weigand. *B. pertussis* strain Tohama 1 and D420 were cultured on Bordet-Gengou (BG) agar (Remel, catalog no. R45232) that was supplemented with 15% defibrinated sheep blood (Hemostat Laboratories, catalog no. DSB500) (69). Bacteria were cultured on BG plates for 48 h at 36°C. *B. pertussis* was transferred from BG plates with polyester swabs (Puritan, catalog no. 22-029-574) and transferred into 20 ml of Stainer-Scholte liquid media (SSM) in new, 125-ml flasks (Thermo Fisher Scientific, catalog no. FB50000125) (70). The liquid cultures were grown for 24 h at 36°C inside a shaking incubator at 180 rpm.

### Intranasal challenge with *B. pertussis*.

Seven-week-old ∼170-g female Sprague-Dawley rats (Charles River, catalog no. 001CD) were used for challenge. *B. pertussis* was grown as described above. The rats were then anesthetized with ketamine and xylazine (50 to 100 and 5 to 10 mg/kg, respectively) and challenged with 10^8^ CFU (100 μl intranasally), administering two 50-μl doses, one in each nostril (Fig. 1). The body weight of each rat was recorded both before challenge and immediately after euthanasia. Rats were euthanized at days 1, 3, 6, 9, and 12 after bacterial challenge. Mock-challenge animals (no bacteria) were administered 100 μl of sterile endotoxin-free PBS (Thermo Fisher Scientific, catalog no. TMS012A) intranasally. Upon euthanasia, blood was collected via cardiac puncture and transferred into EDTA (BD, catalog no. 365974) and serum separation (BD, catalog no. 026897) tubes. Blood collected in the EDTA tube was used for flow cytometric analysis, while blood collected in the serum separation tubes were used to isolate the serum via centrifugation (15,000 × *g* for 3 min) and used for serological and cytokine analysis. Lungs and tracheas were excised and homogenized to determine the bacterial burden. Upon removal, the wet weight of the lungs was recorded. Lungs were then collected in gentleMACS C tubes (Miltenyi Biotec) and homogenized using a gentleMACS dissociator (catalog no. 130-095-927). The trachea was homogenized using a Polytron homogenizer. To determine the bacterial burden in the nares, 2 ml of sterile 1× PBS was flushed through the nares, and then samples were collected for plating. Serial dilutions of the homogenates and nasal collections were plated on BG plates supplemented with ceftibuten (Sigma-Aldrich, catalog no. SML0037) at 10 μg/ml to decrease the growth of normal rat respiratory tract flora.

### Analysis of coughing and bronchiole restriction using whole-body plethysmography.

To quantify respiratory function during infection, we utilized a Buxco FinePointe Whole Body Plethysmograph instrument (WBP; DSI). FinePointe software was used to collect, analyze, and report the breathing data. Every day postchallenge for 12 consecutive days at 5:00 p.m., the rats were placed inside designated chambers to acclimate for 5 min. We chose this time not only due to rats being nocturnal but also because at this time the animals were most awake and active, as confirmed by video camera (data not shown). After acclimation, each rat’s respiratory profile was recorded for 15 min. Each chamber is fitted with a transducer that measured the changes in box flow and airflow of the subject. The chambers were also fitted with a screen pneumotach that allowed airflow in and out of the chamber that can be recorded. Coughs were counted during the designated 15 min, and the enhanced pause (PenH) was calculated to signify bronchiole restriction. Coughs were counted based on large box flow changes of the subject and changes in both humidity and temperature of the air flowing in and out of the subject with classical cough-like waveforms. Cough detection algorithm was applied using a patented fuzzy logic criteria to determine whether the event was a cough (71). When analyzing the number of coughs, each cough in a multicough event was counted individually.

### Histological assessment of the lung and NALT.

Upon euthanasia, the rat skull was excised, and the mandible was removed. The skulls were fixed in 10% formalin (Fisher Scientific, catalog no. SF98-4) for 48 h at 26°C. After fixation, the formalin was removed, and the skulls were frozen at −80°C until decalcification. Skulls were decalcified using Richard-Allan Scientific decalcifying solution (Thermo Scientific, catalog no. 8340-1) at room temperature for 24 to 48 h and then embedded in paraffin. Samples were sectioned and stained with H&E. A BioTek Lionheart Fx was used to scan and image the NALT. ImageJ was used to trace and measure the area of both the left and the right NALT using the images from the Lionheart Fx. The left lobe of the lung was excised and fixed in 10% formalin 48 h at 26°C. The left lobe was then embedded in paraffin and stained with H&E by the WVU Pathology Department. H&E-stained sections were used to characterize and score acute and chronic inflammation of the lung. All scorings were done by a board-certified pathologist (iHisto). Individual scores were based on a standard qualitative scoring criterion: 0, none; 1, minimal (rare); 2, mild (slight); 3, moderate; 4, marked; and 5, severe. Chronic inflammation was characterized by mononuclear infiltrates of the parenchyma, blood vessels, and airway. Acute inflammation scores were assigned due to the presence of neutrophils in the parenchyma, blood vessels, and airway. All examination and scoring were performed blindly since no knowledge of the treatment groups was known.

### Imaging of *B. pertussis* in the lung and nasal cavity.

Detection of *B. pertussis* in the nasal cavity and lung was quantified via IF and confocal imaging. The left lobe of the lung and nasal cavity were preserved and sectioned as described above. Sectioned samples underwent deparaffination and rehydration using xylene and ethanol (70 to 100%). Antigen retrieval was performed by incubating samples in citrate buffer at 98°C for 20 min. Samples were blocked using 5% bovine serum albumin (Fisher Scientific, catalog no. 159008) for 1 h and primarily labeled utilizing a polyclonal rabbit FHA antibody (a gift from Erik Hewlett) diluted in 1× PBS. Secondary labeling occurred utilizing an anti-rabbit IgG conjugated with Texas Red (Fisher Scientific, catalog no. T2767) diluted in 1× PBS. Samples were then covered in mounting media (Prolong Gold Antifade reagent with [4′,6′-diamidino-2-phenylindole], catalog no. 8961). Samples were imaged using a Nikon A1R confocal microscope. Images were analyzed on DAPI channel and at wavelength 650 nm for Texas Red acquisition. Images were acquired using a 100× oil immersion lens (100×/1.40 Nikon Plan APO). To identify any potential differences in IF between the two strains, all IF images were deidentified, and the microcolonies were manually counted blindly by four volunteers with two to three fields of view used per sample.

### Flow cytometry analysis of phagocytes.

Neutrophil recruitment in the blood was evaluated by flow cytometry. Blood samples upon collection were then lysed with PharmLyse buffer (BD Biosciences, catalog no. 555899) for 20 min at room temperature, with slight vortexing throughout. The remaining cells were resuspended in RPMI–10% fetal bovine serum (FBS) to neutralize the lysis buffer, centrifuged five times at 1,000 × *g*, and then washed with RPMI–10% FBS again. Blood was then resuspended in 1% FBS–PBS–5 mM EDTA. Samples were next blocked with anti-CD32 (BD Pharmingen, catalog no. 550270) antibody for 30 min at 4°C. After incubation, the cells were stained with the appropriate antibody markers: CD45-Alexa Fluor 700 (BioLegend, catalog no. 202218), CD161-APC (BioLegend, catalog no. 205606), CD45R-PE-Cy7 (eBioscience, catalog no. 25-0460-82), His48-FITC (eBioscience, catalog no. 11-0570-82), CD43-PE (BioLegend, catalog no. 202812), and CD3-VioGreen (Miltenyi Biotec, catalog no. 130-119-125) (47). After the addition of antibodies, the cells were incubated 1 h at 4°C in the dark. To prepare the lung samples for flow cytometry, the lung homogenate was pushed through a 70-μm-pore-size cell strainer (BioDesign Cell MicroSives, catalog no. N70R), creating a single-cell suspension. The suspension was then centrifuged at 1,000 × *g* for 5 min. After removal of the supernatant, the pellet was resuspended in PharmLyse buffer, and the cells were incubated at 37°C for 2 min. After incubation, the cells were centrifuged at 1,000 × *g* for 5 min, and the supernatant was removed, blocked, and labeled with antibody, as described above. The lung and blood samples were centrifuged at 1,000 × *g* for 5 min, and the pellets were resuspended in 0.4% paraformaldehyde and stored overnight at 4°C. Samples were washed with 1× PBS and resuspended in 1× PBS for analysis. Cell samples were analyzed on a LSR Fortessa, and samples were gated and analyzed using FlowJo v10.

### Lung and serum cytokine and chemokine analysis.

Lung homogenate samples were centrifuged at 19,000 × *g* for 4 min, and the supernatant was removed and stored at −80°C. Quantitative analysis of cytokines in the serum and lung homogenate was performed using a ProcartaPlex multiplex immunoassay kit (Cytokine & Chemokine 22-Plex Rat ProcartaPlex panel, catalog no. EPX220-30122-901) according to the manufacturer’s instructions.

### Serological analysis.

Antibody titers of infected rats were measured by ELISA. B. pertussis-specific ELISA plates were coated with 50 μl of 10^8^ B. pertussis grown, as mentioned above for infection. To measure antibody titers to FHA (Enzo ALX-630-123-0100), PRN (GSK), PT (List Biological Laboratories, catalog no. 180), and ACT (a gift from Erik Hewlett), we coated plates with 50 μl of each antigen at 1 μg/ml. Once coated, the plates were incubated overnight at 4°C. The plates were washed with 1× PBS-Tween 20 and then blocked with 5% skim milk for 2 h at 37°C. The plates were washed again, and the sera from the challenge studies were serially diluted down the ELISA plate, followed by incubation for 2 h at 37°C. After incubation, the plates were washed and coated with 100 μl of secondary goat anti-rat IgG (Southern Biotech, catalog no. 3030-04) and IgM (Southern Biotech, catalog no. 3020-04) antibody at a dilution of 1:2,000 in PBS–5% milk. Once coated, the plate was incubated for 1 h at 37°C. The plates were washed again with PBS-Tween 20, and 100 μl of *p*-nitrophenyl phosphate substate (Thermo Scientific, catalog no. 37620), prepared according to the manufacturer’s instructions, was added. The plate was then developed for 30 min at room temperature to determine the titers of IgG and IgM. A BioTek Synergy H1 microplate reader was used to measure the colorimetric signal of the ELISA plate at *A*_450_. Positive antibody titers were determined as any values that were higher than the baseline. The baseline was set as double the average value of the blank; no serum added to these wells.

### Generating Nightingale Rose plots in Python.

Data from individual mice were averaged in Microsoft Excel and log transformed. Values were formatted in Excel to be compatible with Python. Data were imported from .csv files using the pandas package and plotted using the “Barpolar” representation feature in the plotly.graph_objects module. Samples with a titer of <50 were assigned a value of 0.

### Statistical analysis.

All data were analyzed using GraphPad Prism 7. The minimum number of biological replicates for the challenge studies was 4. For statistical comparisons between multiple groups over the entire course of the infection, a two-way analysis of variance (ANOVA) was used. One-way ANOVA was used for comparisons between groups for each individual day with Tukey’s *post hoc* test. Unpaired Student *t* tests were used for AUC analysis. Specific follow-up statistical tests are annotated in the figure legends.

### Ethics statement.

All studies were performed in accordance with West Virginia University Institutional Animal Care and Use Committee approved protocol 1811019148.6.

### Data availability.

Data requests for figures provided can be addressed to the corresponding author.
