# Supplementary material for: Reinvestigating the Coughing Rat Model of Pertussis To Understand Bordetella pertussis Pathogenesis
Source: Infect Immun. 2021 Nov 16;89(12):e00304-21. doi: 10.1128/IAI.00304-21 (PMC8594615; doi:10.1128/IAI.00304-21)
Supplement: Supplemental file 1 — Supplemental material. Download iai.00304-21-s0001.pdf, PDF file, 1.0 MB [file iai.00304-21-s0001.pdf]

Figure S1

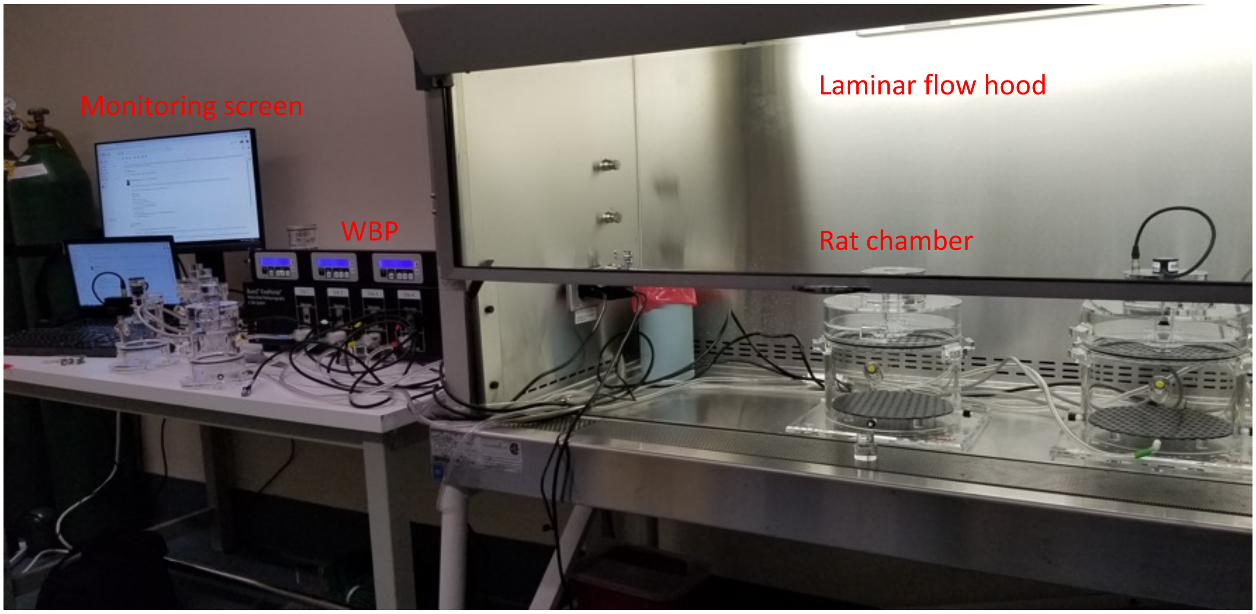

FIG S1 Set up for the whole-body plethysmograph (WBP). Pictured far left on the table is the WBP and computer. Pictured on the right in the bio safety hood are the chambers where the rats are monitored.

Figure S2

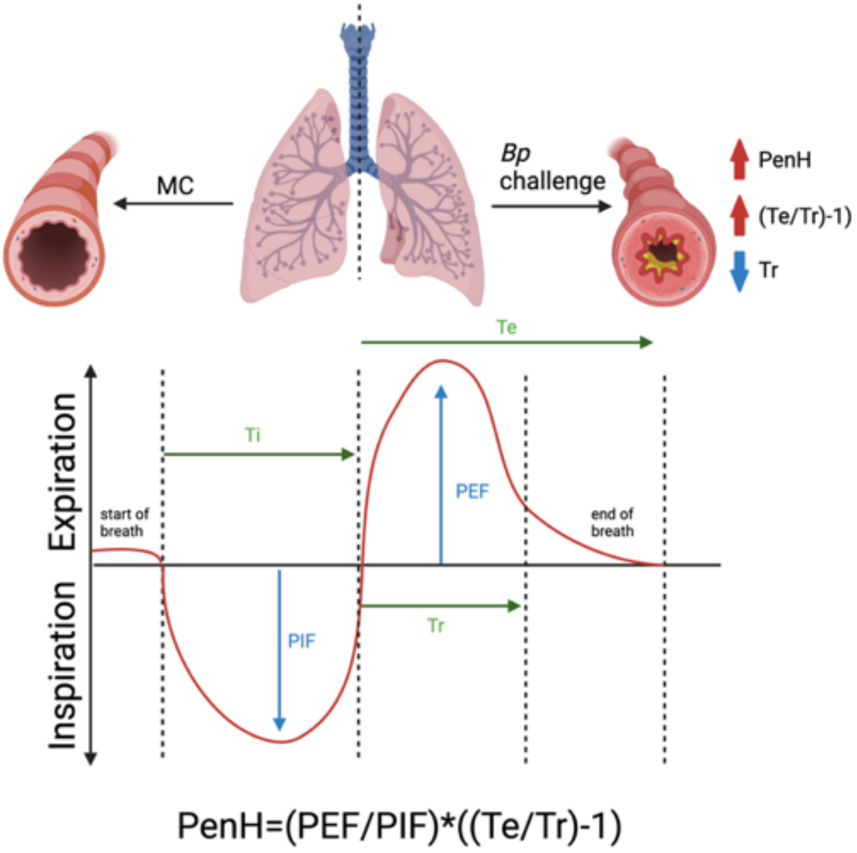

FIG S2 Diagram illustrating Penh during *Bp* infection. Enhanced pause (Penh) is a calculation that is used as a measurement for respiratory distress. Peak expiratory height (PEF) constitutes as the maximum expiratory flow that occurs in one breath; peak inspiratory flow (PIF) is the maximum inspiratory flow that occurs in one breath; Inspiratory time (Ti) the time it takes from the start to end of the inspiration phase of the breath; expiratory time (Te) the amount of time it takes from the start for the expiration to the end of the respiration; relaxation time (Tr) the time it takes the subject to expel certain amount of volume. Following *Bp* challenge, infected rats have an increase in respiratory distress compared to mock challenge rats.

Figure S3

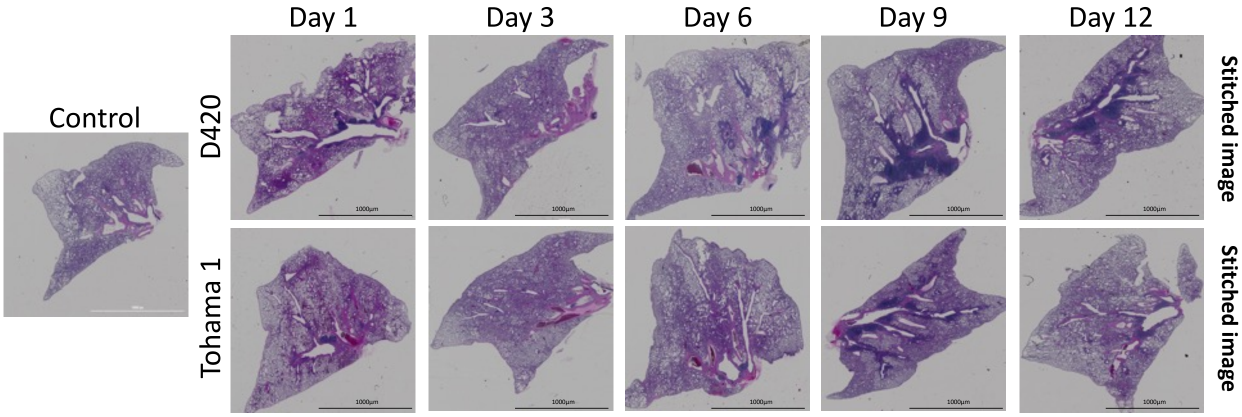

FIG S3 *Bordetella pertussis* infection induces cellular recruitment in the lungs. **(A)** The left lobe of the lung was sectioned and stained with hematoxylin and eosin of rats infected with D420, Tohama 1, or the PBS control. Representative pictures shown are multiple 4x magnification images stitched together for each lung.

Figure S4

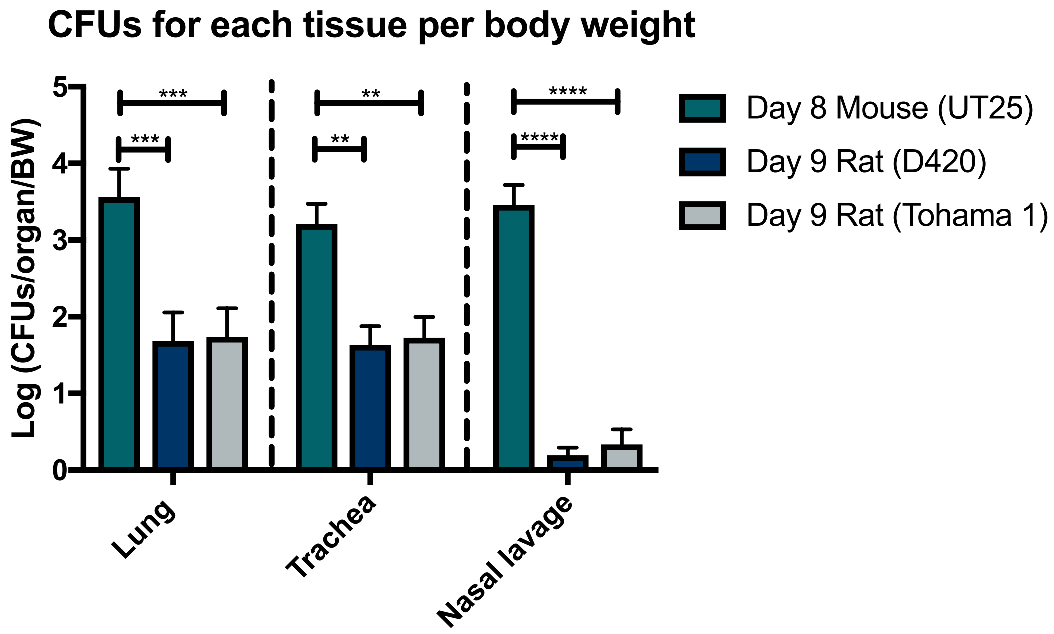

FIG S4 Bacterial colonization comparisons between CD1 mice and Sprague-Dawley rats. To assess the effect of body weight on bacterial colonization, we took CFUs for each respective tissue and divided them by the body weight. CFUs/organ for the CD1 mice came from (Boehm 2018). Results are shown as mean ± SEM ($n = 4$) P values were determined by two-way ANOVA. *$P < 0.05$, **$P < 0.01$, ***$P < 0.001$, ****$P < 0.0001$ compared between groups.

Figure S5

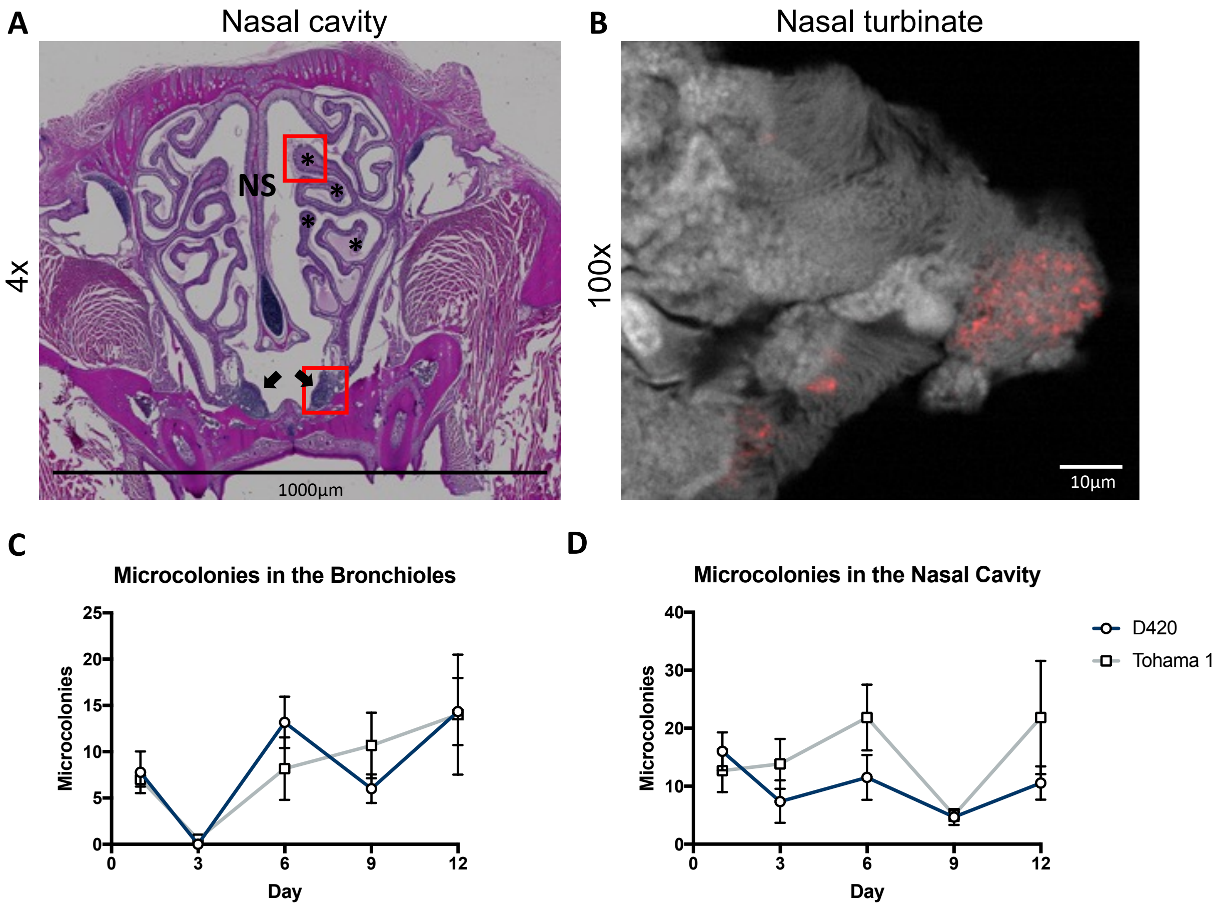

FIG S5 (A) Hematoxylin and eosin staining of the nasal cavity of Sprague-Dawley rat. Arrows represent the nasal associated lymphoid tissue, NS represents the nasal septum, * represents the nasal turbinates. Red boxes indicate areas where the bacteria were found. **(B)** Immunohistochemistry (IHC) staining of *Bordetella pertussis* being captured by the cilia of the nasal cavity. Slides were prepared as described in the Material and Methods over the course of infection. *B. pertussis* was labeled using a polyclonal antibody to FHA and probed with a fluorescently conjugated antibody (Texas-Red). Sections were counterstained with DAPI. **(C-D)** Blinded average microcolony counts of *B. pertussis* in the nasal cavity and the lung. Results shown as mean ± SEM ($n = 3$).

Figure S6

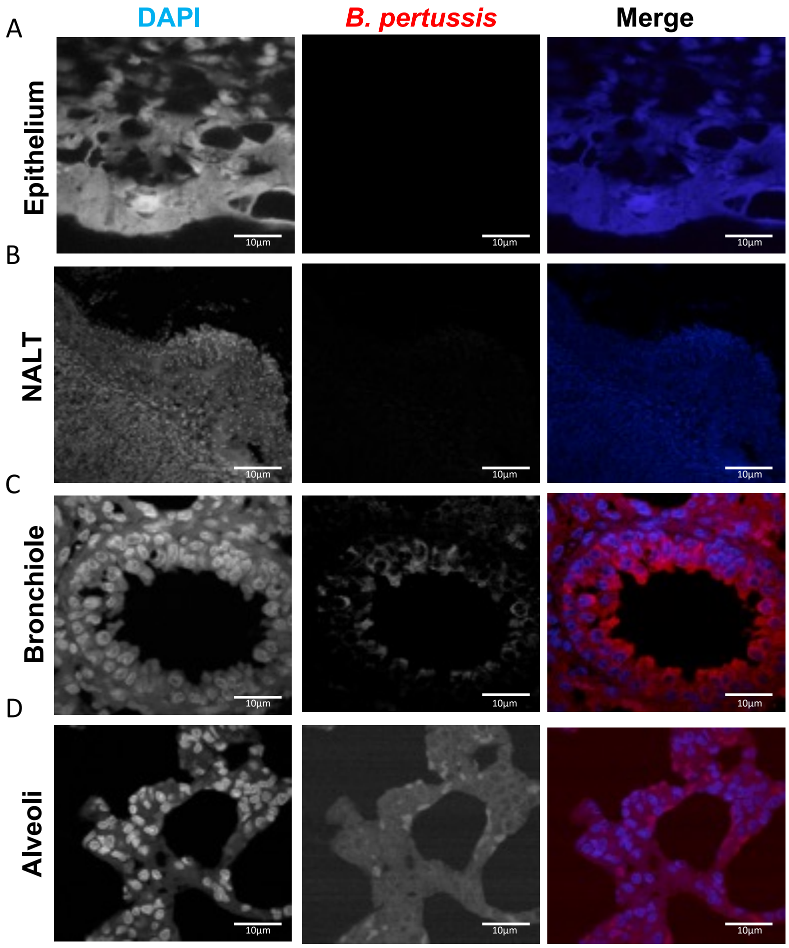

FIG S6 Immunofluorescence (IF) staining of control rat's respiratory tract. *Bp* was probed for using a polyclonal antibody to FHA and counter tagged with a fluorescently conjugated antibody (Texas-Red). Sections were counterstained with DAPI. **(A-D)** Representative images of nasal cavity and lung over the course of infection.

Figure S7

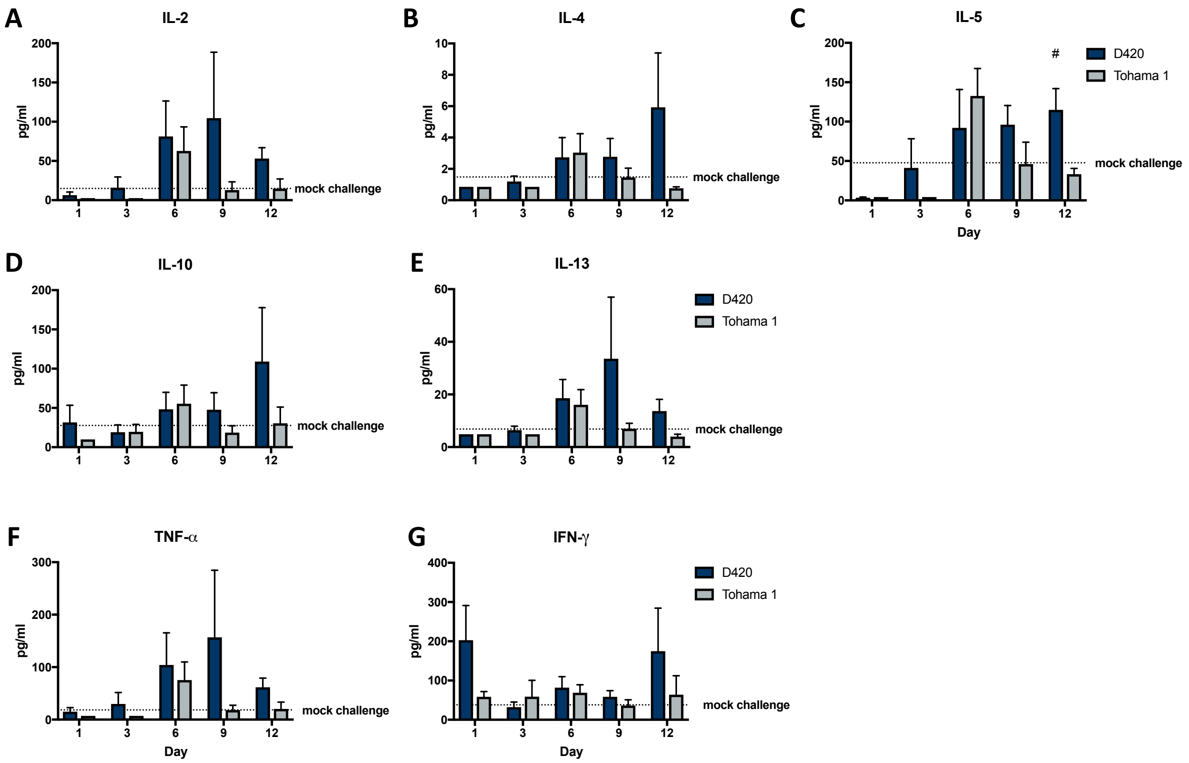

FIG S7 Serum cytokine response during twelve-day *Bordetella pertussis* infection. Cytokines measured include: **(A)** IL-2, **(B)** IL-4, **(C)** IL-5, **(D)** IL-10 **(E)** IL-13, **(F)** TNF-**α**, and **(G)** IFN- γ. Dotted line represents the average cytokine concentration of the mock challenge control group. Results shown as mean ± SEM ($n = 4$). P values were determined by one-way ANOVA followed by Tukey comparison test, #$P < 0.05$ compared between infection groups.

Figure S8

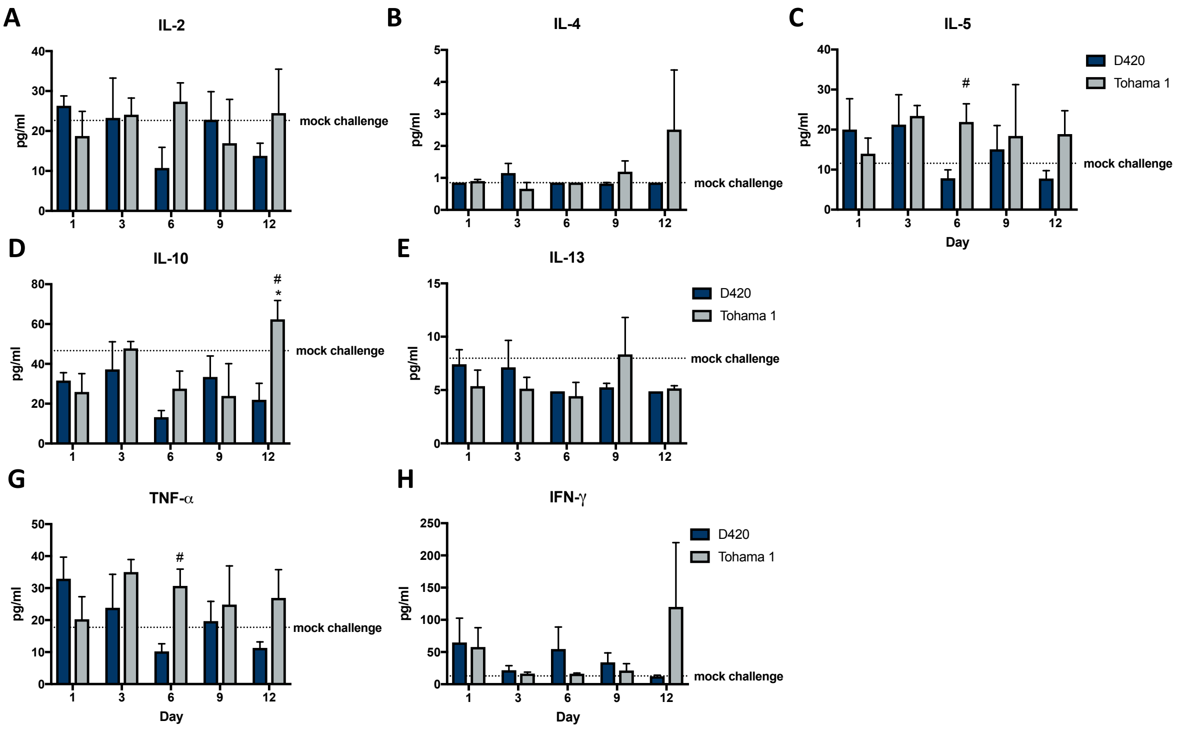

FIG S8 Lung cytokine response during twelve-day *Bordetella pertussis* infection. Cytokines measured include: **(A)** IL-2, **(B)** IL-4, **(C)** IL-5, **(D)** IL-10 **(E)** IL-13, **(F)** TNF-**α**, and **(G)** IFN- γ. Dotted line represents the average cytokine concentration of the mock challenge control group. Results shown as mean ± SEM ($n = 4$). P values were determined by one-way ANOVA followed by Tukey comparison test, #$P < 0.05$ compared between infection groups. *$P < 0.05$ compared to mock challenge.

Figure S9

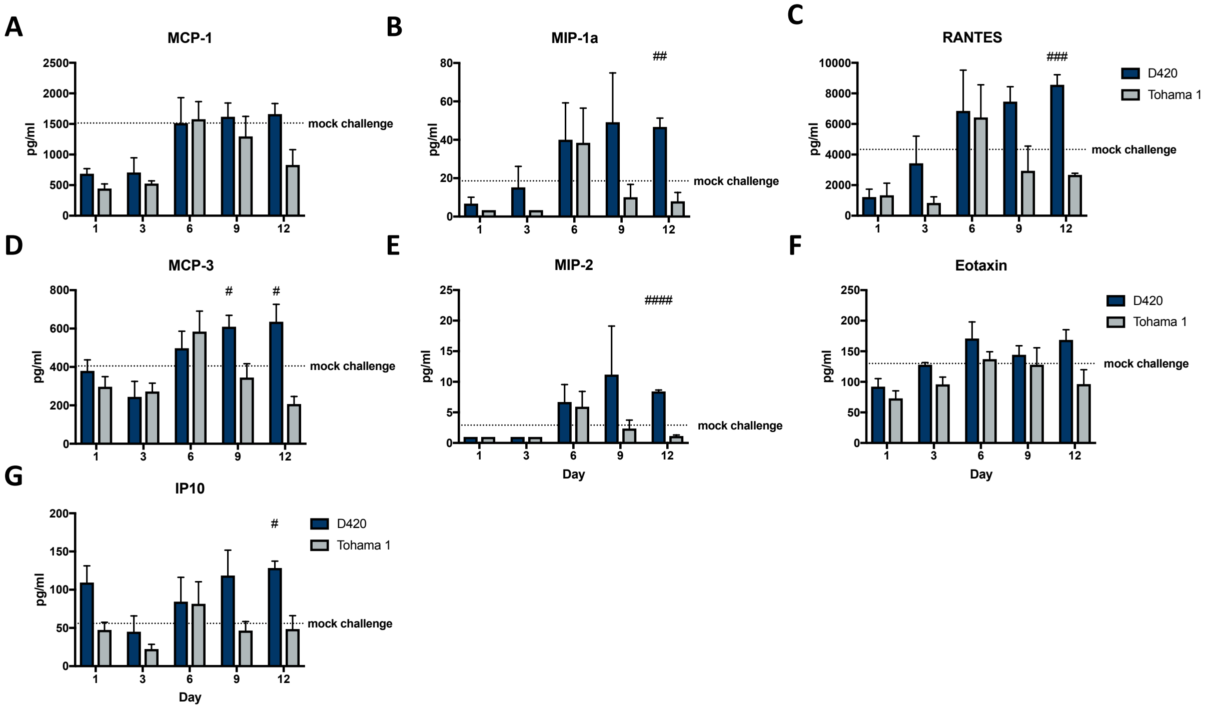

FIG S9 Serum chemokine during twelve-day *Bordetella pertussis* infection. Chemokines measured include: **(A)** MCP-1, **(B)** MIP-1a, **(C)** RANTES, **(D)** MCP-3, **(E)** MIP-2, **(F)** Eotaxin, and **(G)** IP10. Dotted line represents the average cytokine concentration of the mock challenge control group. Results shown as mean ± SEM (n = 4). *P* values were determined by one-way ANOVA followed by Tukey comparison test. #*P* < 0.05, ##*P* < 0.01, ###*P*< 0.001 compared between infection groups.

Figure S10

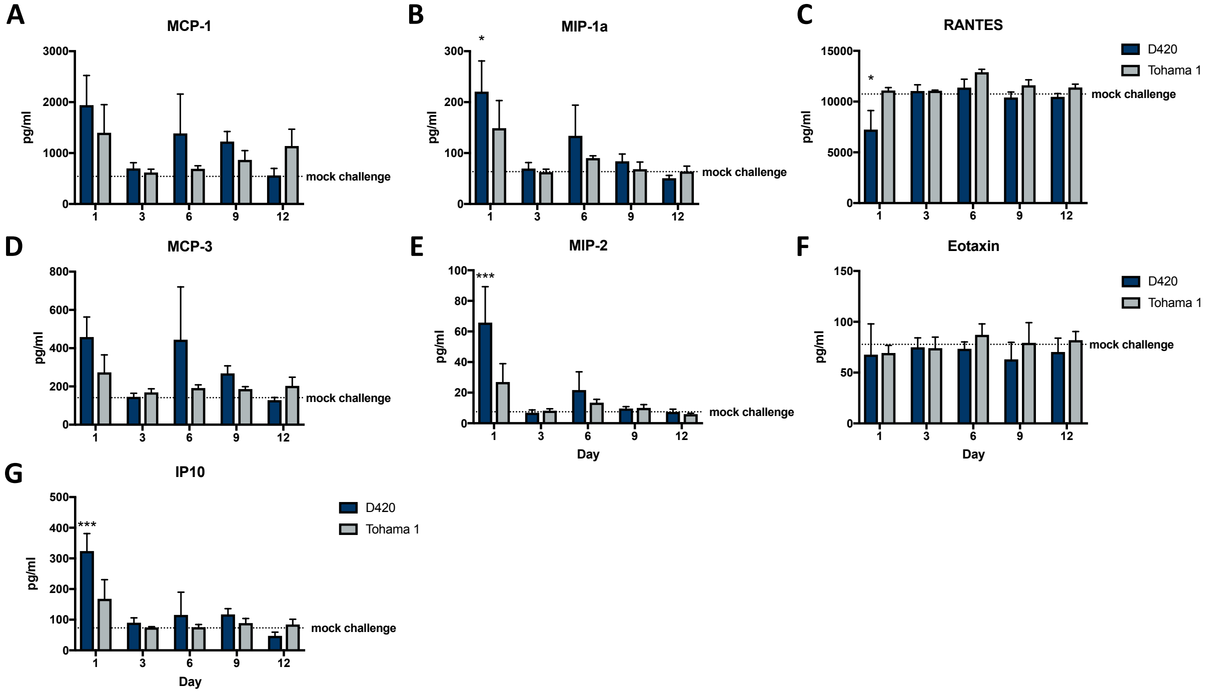

FIG S10 Lung chemokine during twelve-day *Bordetella pertussis* infection. Chemokines measured include: **(A)** MCP-1, **(B)** MIP-1a, **(C)** RANTES, **(D)** MCP-3, **(E)** MIP-2, **(F)** Eotaxin, and **(G)** IP10. Dotted line represents the average cytokine concentration of the mock challenge control group. Results shown as mean ± SEM ($n = 4$). P values were determined by one-way ANOVA followed by Tukey comparison test. *$P < 0.05$, ***$P < 0.001$ compared to mock challenge.

Figure S11

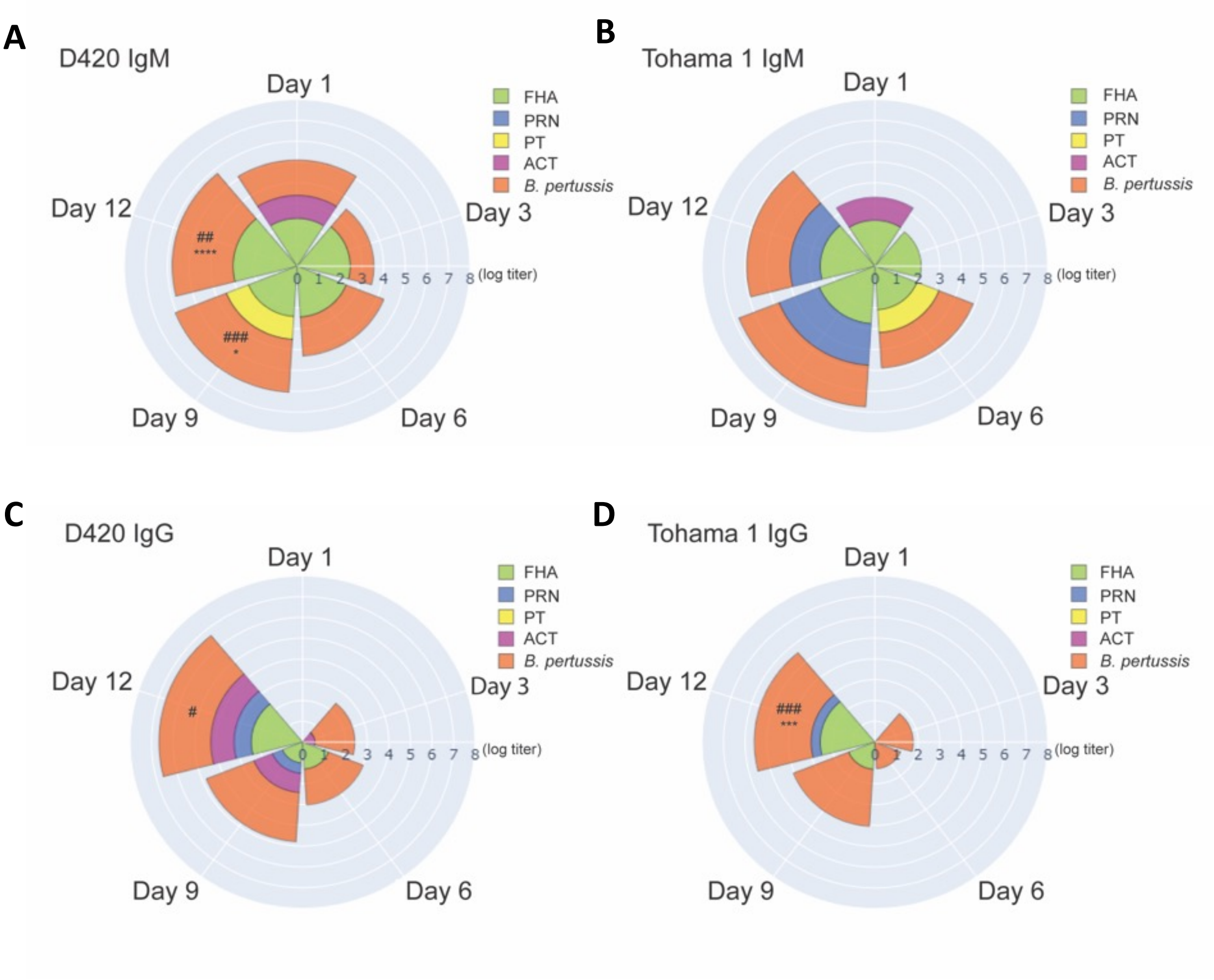

FIG S11 Measurement of serum antibody titers over the course of *B. pertussis* infection. ELISA was used to compare serological responses from rats IN challenge with *B. pertussis*. Total IgM (A-B) and IgG (C-D) serum antibody titers were measured from challenged rats against *Bp*, FHA, PRN, PT, and ACT. ($n = 4$). P values were determined by two-way ANOVA corrected with Bonferroni comparison test, $^{*}P < 0.05$, $^{***}P < 0.001$, $^{****}P < 0.0001$ compared between infection groups. For comparison to mock challenged control group one-way ANOVA was used followed by Tukey comparison test, $^{\#}P < 0.05$, $^{\#\#}P < 0.01$, $^{\#\#\#}P < 0.001$.

Figure S12

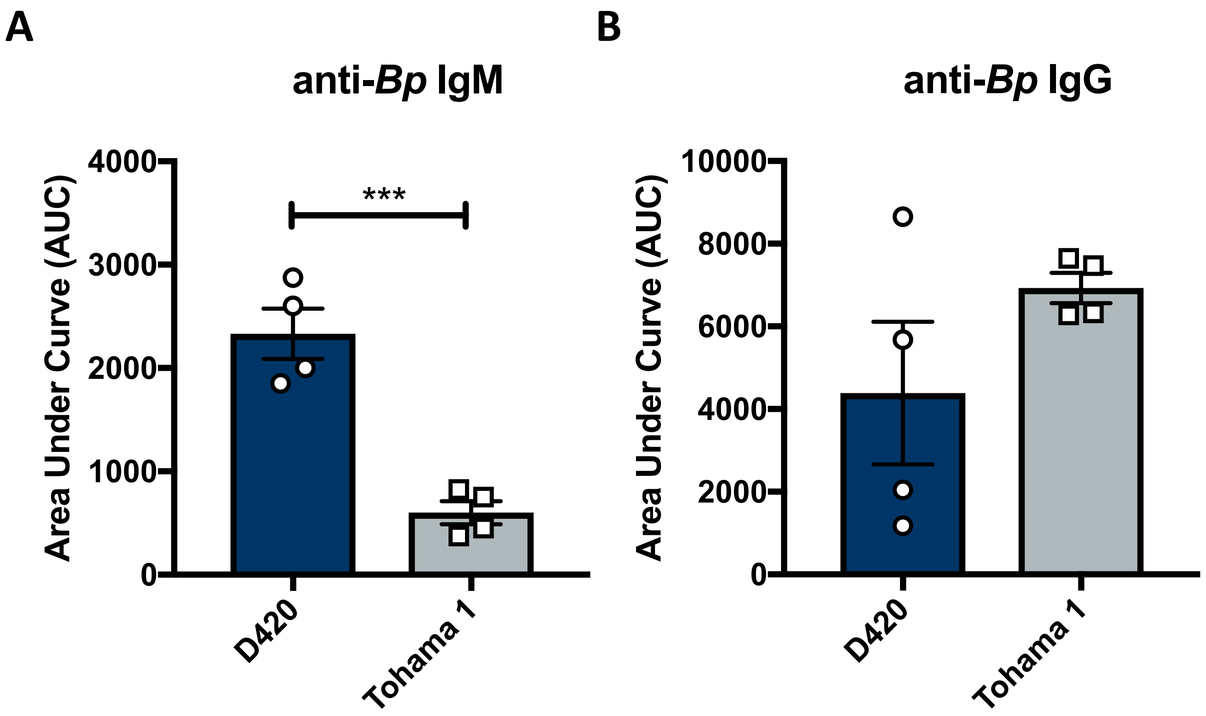

FIG S12 Area under the curve analysis of **(A)** IgM and **(B)** IgG serum antibody titers against *Bordetella pertussis* over the course of infection. Results shown as mean ± SEM ($n = 4$). P values were determined by t-test for area under the curve, ***$P < 0.001$ compared between infection groups.
